# A Two-Compartment Model of VEGF Distribution in the Mouse

**DOI:** 10.1371/journal.pone.0027514

**Published:** 2011-11-08

**Authors:** Phillip Yen, Stacey D. Finley, Marianne O. Engel-Stefanini, Aleksander S. Popel

**Affiliations:** Department of Biomedical Engineering, Johns Hopkins University School of Medicine, Baltimore, Maryland, United States of America; Institut National de la Santé et de la Recherche Médicale, France

## Abstract

Vascular endothelial growth factor (VEGF) is a key regulator of angiogenesis – the growth of new microvessels from existing microvasculature. Angiogenesis is a complex process involving numerous molecular species, and to better understand it, a systems biology approach is necessary. *In vivo* preclinical experiments in the area of angiogenesis are typically performed in mouse models; this includes drug development targeting VEGF. Thus, to quantitatively interpret such experimental results, a computational model of VEGF distribution in the mouse can be beneficial. In this paper, we present an *in silico* model of VEGF distribution in mice, determine model parameters from existing experimental data, conduct sensitivity analysis, and test the validity of the model.

The multiscale model is comprised of two compartments: blood and tissue. The model accounts for interactions between two major VEGF isoforms (VEGF_120_ and VEGF_164_) and their endothelial cell receptors VEGFR-1, VEGFR-2, and co-receptor neuropilin-1. Neuropilin-1 is also expressed on the surface of parenchymal cells. The model includes transcapillary macromolecular permeability, lymphatic transport, and macromolecular plasma clearance. Simulations predict that the concentration of unbound VEGF in the tissue is approximately 50-fold greater than in the blood. These concentrations are highly dependent on the VEGF secretion rate. Parameter estimation was performed to fit the simulation results to available experimental data, and permitted the estimation of VEGF secretion rate in healthy tissue, which is difficult to measure experimentally. The model can provide quantitative interpretation of preclinical animal data and may be used in conjunction with experimental studies in the development of pro- and anti-angiogenic agents. The model approximates the normal tissue as skeletal muscle and includes endothelial cells to represent the vasculature. As the VEGF system becomes better characterized in other tissues and cell types, the model can be expanded to include additional compartments and vascular elements.

## Introduction

Vascular endothelial growth factor (VEGF) belongs to a family of cytokines that play an important role in angiogenesis – the formation of new capillaries from pre-existing vessels. The VEGF family in mammals is composed of VEGF-A, VEGF-B, VEGF-C, VEGF-D and placental growth factor (PlGF). The most well-studied member is VEGF-A (generally referred to as VEGF) that consists of several splice isoforms including VEGF_121_, VEGF_145_, VEGF_165_, VEGF_189_ and VEGF_206_ in humans, where the subscripted number indicates the number of amino acids [1], [2]. The amino acid number for all splice isoforms is one less than in humans for rodent VEGF orthologs. The roles of VEGF_189_ and VEGF_206_ *in vivo* are currently still unclear [3]. Therefore, in our model, we consider the two most abundant isoforms of VEGF-A in the mouse: VEGF_120_ and VEGF_164_.

The tyrosine-kinase receptors of VEGF include VEGFR-1 (Flt-1), VEGFR-2 (Flk-1 or KDR in humans), and VEGFR-3 (Flt-4). VEGFR-1 and VEGFR-2 are the primary receptors for VEGF-A and play a major role in angiogenesis, while VEGFR-3 binds VEGF-C and VEGF-D and plays a major role in lymphangiogenesis. VEGFR-1 and VEGFR-2 are predominantly expressed on endothelial cells; however, these receptors have also been shown to be present on bone marrow-derived cells [4] and other cell types such as neurons and cancer cells. The binding of VEGF-A to VEGFR-2 is believed to be the main signaling pathway for angiogenesis [5]. In addition to the tyrosine-kinase receptors, VEGF-A binds to co-receptor neuropilin-1 (NRP-1). NRP-1 was first found to be expressed on certain tumor and endothelial cell surfaces [5], and has been shown to enhance the binding of VEGF_165_ to VEGFR-2. VEGF can also bind to heparan sulfate proteoglycans in the extracellular matrix (ECM), endothelial cell basement membrane (EBM) and parenchymal cell basement membrane (PBM).

Computational models of VEGF-mediated angiogenesis have been developed to study various aspects of the angiogenic process [6]. A single-compartment model of the human tissue was initially developed to study the kinetic ligand-receptor interactions of multiple VEGF isoforms with endothelial cell surface receptors (VEGFR-1, VEGFR-2, NRP-1) and extracellular matrix binding sites [7]. This model was later expanded to include three compartments, including a tumor compartment, to study tumor angiogenesis [8] and peripheral arterial disease [9]. These compartment models describe spatially averaged VEGF distributions and receptor bindings in the tissue, blood and tumor. However, these models were based on human data and are not immediately applicable to animal data. Mouse animal models have been extensively used to study cardiovascular diseases such as peripheral arterial disease and coronary artery disease [10]. Mouse tumor xenograft models are also commonly used to study different cancers and to develop anti-tumor therapies. Mice are convenient animal models to study human diseases because the overall biology of the mouse is in many respects similar to that of humans, and the two species share many similar characteristics of pathological conditions [11]. Anatomically based three-dimensional models of VEGF-mediated angiogenesis have also been developed to study processes such as endothelial cell migration and proliferation, and capillary sprout formation [12], [13]; however, 3D models are typically limited to smaller scales: microscopic and mesoscopic.

Under physiological conditions, VEGF level in the mouse blood is low (<1.5 pM) [14], possibly as a result of VEGF having a short half-life in this species (3 minutes) [14], [15]. One example of an important mouse study is the work performed by Rudge *et al.,* who describe a high-affinity VEGF antagonist called Aflibercept, engineered to sequester VEGF by forming a complex [16]. The protein is a fully human soluble decoy receptor made by fusing the second Ig domain of human VEGFR-1 to the third Ig domain of human VEGFR-2 with the constant region (Fc) of human IgG1 [17]. Aflibercept, known commercially as “VEGF Trap,” forms an inert complex with VEGF, and the complex has a much longer half-life than unbound VEGF. This anti-VEGF agent is in Phase II studies as an anti-tumor angiogenesis therapy [17]. VEGF Trap was tested on mice bearing mouse tumors as well as mice bearing human tumors. It was found that the mice bearing mouse tumors did not have a significantly higher level of complex in the blood than non-tumor bearing mice, implying that tumor-derived VEGF constituted only a small fraction of total body VEGF or circulating bioavailable VEGF in those mice. Also, since the concentration of the VEGF/VEGF Trap complex can be readily assayed in the blood, Rudge and coworkers used the concentration of the complex to calculate the production rates of VEGF by the host and by the tumor. For mice not bearing tumors, the authors noted that the estimated production rate of VEGF was high compared to previous estimates [16].

Due to the large number of experimental studies performed in mice, including the work of Rudge *et al.*, and given the vast amount of experimental data available from these studies, we have developed a whole-body computational model of VEGF distribution in the mouse, expanding our previous models of VEGF distribution in human [8], [18]–[20]. In this model we take into account VEGF receptor expression on both luminal and abluminal surfaces of endothelial cells, and expression of neuropilin-1 on endothelial cells and myocytes. Parameter estimation was performed to fit the model to available experimental data. Sensitivity analysis was performed to investigate the role of parameters including VEGF secretion rate, VEGF plasma clearance rate, and microvascular permeability to VEGF. Computational modeling of the VEGF system in the mouse provides parameter estimates that are difficult or impossible to extract purely experimentally. In particular, by fitting our model simulation results to the experimental data from Rudge *et al.,* we estimated the value of the endogenous VEGF secretion rate by the myocytes. The mouse model of VEGF distribution can be used in tandem with the previously developed human models to compare the two systems and to scale-up pro- and anti-angiogenic therapeutics for translation from mouse studies (preclinical studies) to human studies (clinical trials).

## Materials and Methods

### Computational model

**Figure 1:**
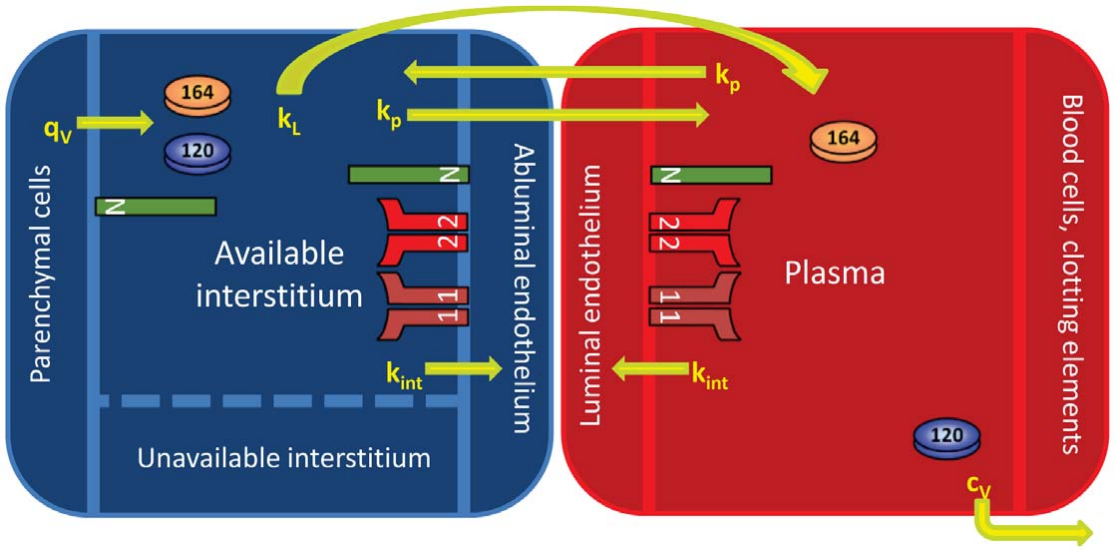
Two compartment model. The model is divided into the tissue and blood compartments. VEGF_120_ and VEGF_164_­ are secreted by the parenchymal cells (myocytes) into the available interstitial space at rate *q_v_*. VEGFR-1, VEGFR-2 and NRP-1 are localized on the luminal and abluminal surfaces of the endothelial cells. NRP-1 is also found on the myocyte cell surface. Inter-compartmental transport includes lymphatic drainage (*k_L_*) and microvascular permeability (*k­_p_*). Receptors and VEGF/receptor complexes on endothelial cells and myocytes can be internalized (*k_int_*). VEGF is removed from the blood compartment via plasma clearance (*c_v_*).

**Figure 2:**
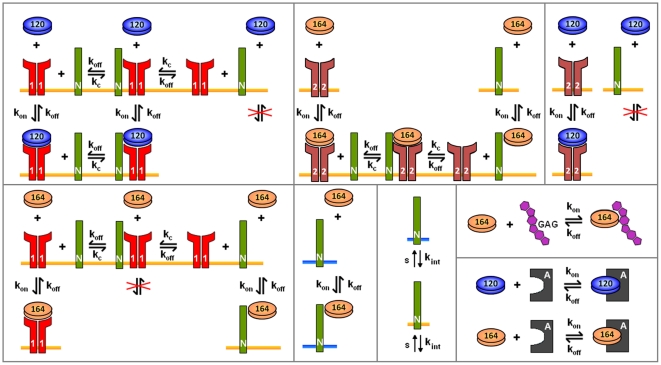
Molecular interactions. The binding interactions of VEGF_120_ and VEGF_164_ are different. VEGF­_120_ binds to VEGFR-1 and VEGFR-2 but not to NRP-1. VEGF_164_ binds to VEGFR-1, VEGFR-2, NRP-1, and glycosaminoglycan (GAG) chains in the extracellular matrix. In simulations where the anti-VEGF agent (VEGF Trap) is added, both isoforms bind to the anti-VEGF agent to form a complex. Binding and unbinding of VEGF to receptors are denoted as *k_on_* and *k_off_*, respectively. *k_c_* denotes the coupling of NRP-1 and VEGFR-1 and of NRP-1 to VEGFR-2. While only the internalization of NRP-1 is shown, all VEGF receptors and complexes can be internalized at a rate *k_int_*. Similarly, while only the insertion of NRP-1 is shown, VEGFR-1 and VEGFR-2 also appear via insertion at a rate *s*. The blue bar is used to distinguish NRP-1 expressed on the myocytes from NRP-1 on the endothelial cells (orange bars).

The basis of this mouse model is the human model developed by Stefanini and co-workers that was used to explore the VEGF distributions in humans in health and disease [8], [18], [19], [21]; the model is significantly expanded as explained below. The mouse is divided into the blood compartment and tissue compartment (Figure 1). As an approximation, the tissue compartment is represented by skeletal muscle that comprises the majority of its mass (43% of total body mass [22]). To build the tissue compartment, the skeletal muscle is represented as cylindrical fibers (myocytes) aligned in parallel with cylindrical microvessels dispersed between the muscle fibers. The space between the muscle fibers and microvessels is designated as the interstitial space, which is itself composed of the basement membranes of the parenchymal cells/myocytes (PBM) and endothelial cells (EBM), in addition to the extracellular matrix (ECM). VEGF is secreted by the myocytes into the interstitial space where it can bind to VEGF receptors VEGFR-1, VEGFR-2, and NRP-1 on the abluminal surface of the endothelial cells, as well as to glycosaminoglycan chains (GAG) in the basement membranes and extracellular matrix. The binding of VEGF to NRP-1 on the surface of the myocytes is also included. VEGF can be transported to the blood via the lymphatics at a rate *k_L_* and can be exchanged between the blood and interstitium via microvascular permeability at a rate *k_p_*. VEGF in the blood can bind to receptors on the luminal surface of the endothelial cells and can be removed via plasma clearance at a rate *c_V_*. Receptors and VEGF/receptor complexes can be internalized at a rate *k_int_.* Figure 2 summarizes the molecular interactions of VEGF_120_ and VEGF_164_ with their receptors and with GAG chains in the PBM, EBM, and ECM.

In our model, we include an anti-VEGF agent that can bind to and form a complex with VEGF in both the blood and tissue. The unbound anti-VEGF agent and the complex are also subject to intercompartmental transport via permeability and lymphatic drainage, and can also be cleared from the blood. The molecular interactions between the two VEGF isoforms and the anti-VEGF agent are illustrated in Figure 2.

We incorporate pore theory in modeling the interstitial space to reflect the available volume for VEGF to diffuse. The VEGF molecules are free to diffuse in the available interstitial fluid volume, denoted *K_AV_*, which is defined as the available fluid volume (*U_AV_*) divided by the total tissue volume (*U*). Based on the geometry of the pores in the basement membranes and extracellular matrix, the partition coefficient and available fluid volume can be calculated as follows:
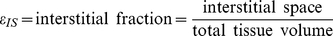


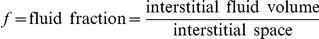


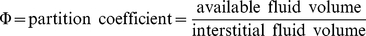


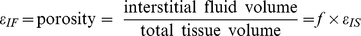


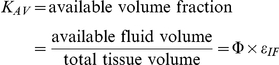



### Equations

The model is fully described with forty coupled ordinary differential equations (ODEs) including 24 molecular species in the tissue and 16 molecular species in the blood, representing a total of 94 chemical reactions. The complete set of equations and chemical reactions are presented in Text S1. Equations 1–3 show the ODEs for VEGF_164_, VEGF_120_, and the anti-VEGF agent (*A*) in the tissue (denoted by superscript or subscript *N* for normal tissue), and equations 4-6 show the ODEs for these same molecular species in the blood (denoted by superscript or subscript *B*).
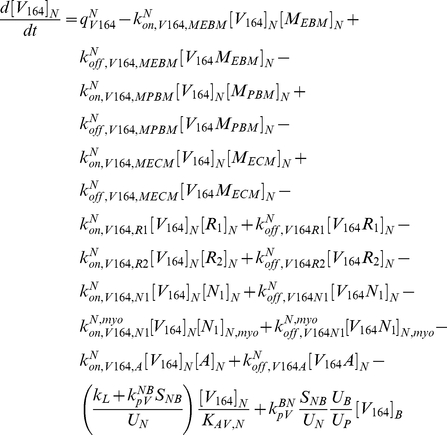
(1)

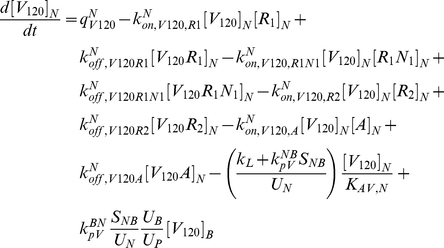
(2)

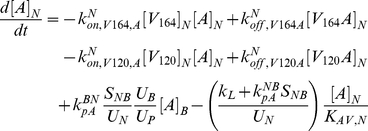
(3)

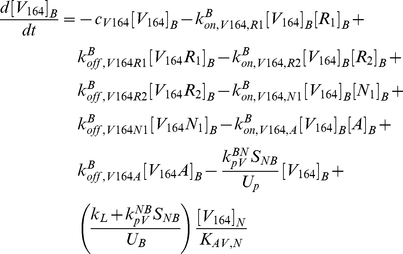
(4)

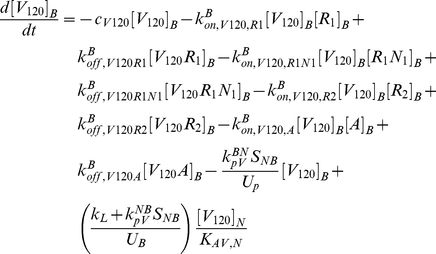
(5)

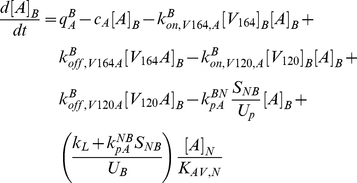
(6)


In these equations, *q_V_* represents the secretion of VEGF in the tissue, and *q_A_* represents the injection rate of the anti-VEGF agent in the blood. Transport parameters include plasma clearance (*c_V_*), lymphatic drainage (*k_L_*), and microvascular permeability (*k_p_*). *k_on_* and *k_off_* represent the kinetic binding and unbinding rates of VEGF with the receptors and with the anti-VEGF agent. Geometric parameters include the total surface area of microvessels at the blood/tissue interface (*S_NB_*), the total tissue volume (*U_N_*), the total blood volume (*U_B_*), and the total plasma volume (*U_p_*). *K_AV,N_* represents the available volume fraction for VEGF in the tissue.

### Numerical implementation

The forty differential equations were implemented in MATLAB® (v7.10.0.499 R2010a, Mathworks®) using the SimBiology® toolbox and the simulations were run on a laptop PC. All simulations were performed using the sundials solver routine with an absolute tolerance of 10^−20^ and a relative tolerance of 10^−5^.

### Model parameters

The overall model is parameterized for a 25-gram mouse and is not currently strain specific. Model parameters are summarized in Tables 1, 2, 3, 4, and 5.

The formulation of the two-compartment mouse model follows the scheme we previously applied to the human model [7]. That is, we first begin by determining parameters of the whole mouse, such as the mass and blood volume. The whole-mouse is then divided into the blood and tissue compartments. Then the blood and tissue compartments are further characterized by parameters detailing the plasma volume and tissue geometry, respectively.

The parameters describing the whole mouse are presented in Table 1. The volume of blood for a mouse is 6–8 mL per 100 g body weight [23], yielding 1.75 mL of blood for a 25-gram mouse. Plasma volume used in our model is 0.85 mL, based on 3.42 mL of plasma per 100 g body weight [24]. Hence, the plasma volume accounts for 49% of the blood volume. We consider the volume of the tissue to be the difference between the total volume of the mouse and the volume of the blood. The density of whole blood is 1.002 g/cm^3^ [25]; thus the mass of blood is calculated to be 1.75 g. This yields 23.25 g as the mass of the tissue, and using 1.06 g/cm^3^ as the density of skeletal muscle [26], we calculate the volume of the tissue to be 21.93 cm^3^.

**Table 1:** Whole-body mouse parameters mouse.

	Value	Unit	Reference
**Mass of mouse**	25	g	[16]
**Blood volume**	1.75	mL	[23]
**Plasma volume**	0.85	mL	[24]
**Plasma fraction**	0.49	-	Calculated (see manuscript)
**Mass of blood**	1.75	g	Calculated (see manuscript)
**Mass of tissue**	23.25	g	Calculated (see manuscript)
**Tissue volume**	21.93	cm^3^	Calculated (see manuscript)

To model the tissue compartment, we used many properties of the mouse gastrocnemius muscle since this muscle is extensively studied and characterized (Table 2). However, not all necessary parameter values for the mouse gastrocnemius are available and we had to use available parameters from other muscles. The interstitial space in the mouse pectoral muscle is 14.4 µL/100 mg wet weight [27] and thus the fraction of tissue that is interstitial space is calculated to be 0.15, using 1.06 g/mL as the density of skeletal muscle [26]. The capillary density of the mouse gastrocnemius has been measured to range from 300 to 1,700 capillaries/mm^2^ [28]–[33]. This wide range is due to different factors such as mouse strain, age and exercise level.

**Table 2:** Geometric parameters of mouse gastrocnemius muscle.

	Value	Unit	Reference
**Capillary density**	650	#/mm^2^	[28]–[33]
**Capillary/fiber ratio**	1.95	-	[28]–[33]
**Fiber cross-sectional area (FCSA)**	2,500	µm^2^	[28]–[33]
**Fiber density**	33,333	#/cm^2^	[28]–[33]
**Luminal capillary diameter**	5.25	µm	[34]–[37]
**Capillary wall thickness**	0.5	µm	[78]
**External capillary diameter**	6.25	µm	Calculated (see manuscript)
**Capillary volume**	1.99%	cm^3^/cm^3^ tissue	Calculated (see manuscript)
**Capillary perimeter**	21.77	µm	Calculated (see manuscript)
**Capillary surface area**	155.68	cm^2^/cm^3^ tissue	Calculated (see manuscript)
**Fiber volume**	82.74%	cm^3^/cm^3^ tissue	Calculated (see manuscript)
**Fiber perimeter**	214.10	µm	Calculated (see manuscript)
**Fiber length**	8.56	µm	Calculated (see manuscript)
**Single myonuclear domain (SMND) surface area**	1.83×10^−5^	cm^2^	Calculated (see manuscript)
**EBM thickness**	154	nm	[46]
**PBM thickness**	154	nm	[46]
**BM pore size**	7	nm	[49]
**ECM pore size**	66	nm	[50]
**EBM volume**	0.002014	cm^3^/cm^3^ tissue	Calculated (see manuscript)
of which available to VEGF	0.001983	cm^3^/cm^3^ tissue	Calculated (see manuscript)
**PBM volume**	0.009123	cm^3^/cm^3^ tissue	Calculated (see manuscript)
of which available to VEGF	0.008986	cm^3^/cm^3^ tissue	Calculated (see manuscript)
**ECM volume**	0.141463	cm^3^/cm^3^ tissue	Calculated (see manuscript)
of which available to VEGF	0.121419	cm^3^/cm^3^ tissue	Calculated (see manuscript)
**K_AV_**	0.113117	cm^3^/cm^3^ tissue	Calculated (see manuscript)

Because we first constrained the fractional volume of interstitial space, other tissue parameters needed to be adjusted accordingly to yield reasonable values for fractional volumes of muscle fibers and blood. Particularly, the capillary density, capillary/fiber ratio, and fiber cross-sectional area determine the remaining parameters required to characterize the mouse model. We used a capillary density of 650 capillaries/mm^2^, a capillary/fiber ratio of 1.95, and a fiber cross-sectional area of 2,500 µm^2^, which are consistent with experimental measurements [28]–[33]. This then yields a fiber density of 333 fibers/mm^2^ and a fiber fractional volume of 0.83. This leaves a blood fractional volume of 0.014, which requires the luminal capillary diameter to be 5.25 µm. Capillary diameters in the mouse have been measured to range from 3.6 µm in the calf muscle to 5.9 µm in the skin [34]–[37]. Assuming the capillary wall thickness to be 0.5 µm, the external capillary diameter is then 6.25 µm and the capillary volume is calculated to be 2% of the total tissue volume. The capillary cross-sectional area is 30.67 µm^2^ and using a capillary perimeter to cross-sectional area correction factor of 1.23 [38], [39], the capillary perimeter is calculated as 21.77 µm. A capillary surface area correction factor of 1.1 [7] yields the capillary surface area of 155.68 cm^2^/cm^3^ tissue. The abluminal and luminal surface areas of one endothelial cell are each taken to be 1,000 µm^2^ [40].

The fiber volume fraction is corrected to account for the capillary wall thickness and is calculated to be 82.74%. Using a fiber perimeter correction factor of 1.21 [28], the fiber perimeter is calculated to be 214.10 µm and the surface area is then 713.68 cm^2^/cm^3^ tissue. The gastrocnemius is a mixed muscle; however, it has been reported that the outer zone comprises most of the mouse gastrocnemius, and that it is comprised of 94% type IIA fibers [41]. Hence in the model, we base the fiber geometry on type IIA fibers. In particular, the myonuclear domain size for mouse type IIA fibers is 21,400 µm^3^/myonucleus [42]. Using this estimate and a fiber cross-sectional area of 2,500 µm^2^, the calculated length of one muscle fiber myonuclear domain is 8.56 µm. The surface area of a myonuclear domain is then calculated to be 1.83×10^−5^ cm^2^. Although the calculated muscle fiber length is shorter than that calculated for other animals and muscle types, similar measurements have been observed experimentally. For example, in the rat gastrocnemius, type IIA muscle fibers were found to have approximately 50 nuclei/mm (myonuclear fiber length of 20 µm) and cross-sectional areas of 2500 µm^2^ [43]. In the mouse, the extensor digitorum longus (EDL) and soleus have approximately 40 and 60 nuclei/mm (myonuclear fiber lengths of 17 and 25 µm), respectively [44]. However, the EDL and soleus muscles are primarily composed of type I and type IIB fibers, respectively [45], and have cross-sectional areas of less than 2,000 µm^2^ [44].

The interstitial space is assumed to be composed of the extracellular matrix (ECM), parenchymal basement membrane (PBM) and endothelial basement membrane (EBM). Although VEGF is able to diffuse in the interstitial space, part of this volume is inaccessible to VEGF. The thicknesses of the basement membranes are 154 nm [46], yielding volume fractions of 0.0020 and 0.0091 cm^3^/cm^3^ tissue for the EBM and PBM, respectively. The remaining interstitial space volume of 0.14 cm^3^/cm^3^ tissue is taken to be the extracellular matrix. These three elements of the interstitial space are assumed to have a solid fraction composed primarily of collagen, which is unavailable to VEGF, and a fluid fraction that is accessible to VEGF. The fraction of body weight that is composed of collagen in the mouse is estimated to be 2.5% [47]. Using a density of 1.41 g/cm^3^ for collagen [48], the total volume of collagen in the interstitial space in a 25-gram mouse is 0.443 cm^3^. The ratio of basement membrane collagen to total body collagen is 0.0083 [47]. Using this ratio, the total collagen in the ECM, EBM, and PBM is then calculated to be 0.4396 cm^3^, 0.0007 cm^3^, and 0.0030 cm^3^, respectively. Hence, the non-collagen fractions of the ECM, EBM, and PBM volumes are 86%, 98%, and 98%, respectively.

We further consider pores in the ECM, EBM, and PBM, which may be inaccessible to freely diffusible molecules in the interstitial space. The EBM pore size for rat brain capillaries has been measured to be 7 nm [49] and the ECM pore size is 66 nm in humans [50]. With these pore sizes, the partition coefficients of the EBM and PBM are 0.35, and the partition coefficient for the ECM is 0.90 [51]. The available space for the ECM and basement membranes for VEGF to diffuse is then calculated as the product of the volume, fluid fraction, and partition coefficient. The total available space in the interstitial space (*K_AV_)* is then calculated to be 0.113 cm^3^/cm^3^ tissue.

Receptor densities and ECM binding site densities are listed in Table 3. VEGF receptors VEGFR-1, VEGFR-2, and co-receptor NRP-1 are assumed to be evenly distributed along both the luminal and abluminal surfaces of the microvessels [19], [52]. Furthermore, NRP-1 is distributed on the surface of myocytes. Based on quantitative flow cytometry measurements in the mouse gastrocnemius *in vivo*, the concentrations of VEGFR-1, VEGFR-2 are 1,050 and 700 dimerized receptors per endothelial cell, respectively (Imoukhuede and Popel, unpublished observations). The number of NRP-1 dimers per endothelial cell is approximately 35,000 based on *in vitro* studies [53]. Additionally, *in vitro* characterization of human quadriceps muscles estimated 35,000 neuropilin-1 dimers per myonuclear domain (Imoukhuede and Popel, unpublished observations). To our knowledge, there are currently no quantitative measurements *in vivo* of NRP-1 on endothelial and myocyte cell surfaces.

**Table 3:** Receptor densities.

	Model parameters		Tissue parameters		
	Value	Unit	Value (Tissue)	Value (Blood)	Unit
**VEGFR-1**	1,050	dimers/EC			pmol/cm^3^ tissue
Luminal EC	525	dimers/EC	-	1.70×10^−1^	pmol/cm^3^ tissue
Abluminal EC	525	dimers/EC	1.36×10^−2^	-	pmol/cm^3^ tissue
**VEGFR-2**	700	dimers/EC			pmol/cm^3^ tissue
Luminal EC	350	dimers/EC	-	1.13×10^−1^	pmol/cm^3^ tissue
Abluminal EC	350	dimers/EC	9.07×10^−3^	-	pmol/cm^3^ tissue
**Neuropilin-1**	35,000	dimers/EC			pmol/cm^3^ tissue
Luminal EC	17,500	dimers/EC	-	5.67	pmol/cm^3^ tissue
Abluminal EC	17,500	dimers/EC	4.53×10^−1^	-	pmol/cm^3^ tissue
Myocytes	35,000	dimers/myocyte	2.26	-	pmol/cm^3^ tissue
**ECM binding density**	0.75	µM	82.50	-	pmol/cm^3^ tissue
**EBM binding density**	13	µM	9.02	-	pmol/cm^3^ tissue
**PBM binding density**	13	µM	40.95	-	pmol/cm^3^ tissue

EC  =  endothelial cell.

Conversions:

Abluminal EC receptors: 2.59×10^−5^ (pmol/cm^3^ tissue)/(dimers/EC).

Luminal EC receptors: 3.24×10^−4^ (pmol/cm^3^ tissue)/(dimers/EC).

Myocyte receptors: 6.46×10^−5^ (pmol/cm^3^ tissue)/(dimers/myocyte).

ECM: 1.10×10^8^ (pmol/cm^3^ tissue)/M.

EBM: 6.94×10^5^ (pmol/cm^3^ tissue)/M.

PBM: 3.15×10^6^ (pmol/cm^3^ tissue)/M.

It is known that VEGF_164_ binds to the glycosaminoglycan (GAG) chains of the heparan sulfate proteoglycans in the extracellular matrix [54]; however to our knowledge, there are currently no direct experimental measurements of binding affinities of VEGF to the ECM. Based on the binding affinities of basic fibroblast growth factor to GAG chains in the ECM, which are assumed to be similar to VEGF binding affinities, the ECM, PBM, and EBM binding site densities are taken to be 0.75, 13, and 13 µM respectively [55].

Transport parameters for VEGF, anti-VEGF, and the VEGF/anti-VEGF complex are listed in Table 4. In mice, the half-life of VEGF in the circulation has been reported to be approximately 3 min [15], [16]. The VEGF clearance rate in plasma is then 0.23 min^-1^, which is equal to ln(2)/half-life. VEGF is assumed to be a 45-kDa globular molecule and its microvascular permeability is taken to be 4.0×10^−8^ cm/s in accordance with previous models based on the molecular weight [8], [18], [19], [21], [56], [57]. In previous models, the microvascular permeabilities for molecules were determined based on their molecular weights and Stokes-Einstein radii [18], [56], [57]. In particular the permeability for bevacizumab, a VEGF antibody, was 3×10^−8^ cm/s based on a molecular weight of 150 kDa [8]. VEGF Trap, which has a molecular weight of 115 kDa [58], is taken to have a comparable size compared to bevacizumab. Thus the permeability of VEGF Trap is also chosen to be 3×10^−8^ cm/s. The VEGF secretion rate, lymphatic drainage rate, and clearance rates of anti-VEGF and the VEGF/anti-VEGF complex were determined using experimental data and are discussed in the “Free parameters” subsection of the Results.

**Table 4:** Transport parameters.

	Value	Unit	Reference
**VEGF secretion rate (total)** *	0.0680	molecules/cell/s	see manuscript
VEGF_164_ secretion rate	0.0626	molecules/cell/s	see manuscript
VEGF_120_ secretion rate	0.0054	molecules/cell/s	see manuscript
**Lymphatic drainage** *	7.00×10^−6^	cm^3^/s	[61], [62]
**Permeability**			
VEGF	4.00×10^−8^	cm/s	[18]
anti-VEGF & VEGF/anti-VEGF complex	3.00×10^−8^	cm/s	[8]
**Clearance**			
VEGF	0.23	min^−1^	[15]
anti-VEGF*	8.86×10^−4^	min^−1^	[63]
VEGF/anti-VEGF complex*	2.79×10^−4^	min^−1^	[63]

*Optimized parameter values.

The kinetic parameters for the binding and unbinding of VEGF to VEGFR-1, VEGFR-2, NRP-1, and GAG chains are listed in Table 5 and are identical to those used in previous models [7], [10], [21]. Kinetic parameters for the binding and unbinding of VEGF to the anti-VEGF agent were fitted parameters and are discussed in the “Free parameters” subsection of the Results. Because VEGF Trap is the anti-VEGF agent used in the model presented here, VEGF Trap and anti-VEGF are used interchangeably throughout this text.

**Table 5:** Kinetic parameters of VEGF and anti-VEGF.

	Measured parameters		Tissue parameters		
**VEGF binding to VEGFR-1**	Value	Unit	Value (Tissue)	Value (Blood)	Unit
k_on_	3.00×10^7^	M^−1^s^−1^	2.65×10^−1^	6.15×10^−2^	(pmol/cm^3^ tissue)^−1^s^−1^
k_off_	1.00×10^−3^	s^−1^			
K_d_	33	pM	3.73×10^−3^	1.61×10^−2^	pmol/cm^3^ tissue
**VEGF binding to VEGFR-2**					
k_on_	1.00×10^7^	M^−1^s^−1^	8.84×10^−2^	2.05×10^−2^	(pmol/cm^3^ tissue)^−1^s^−1^
k_off_	1.00×10^−3^	s^−1^			
K_d_	1.00×10^2^	pM	1.13×10^−2^	4.88×10^−2^	pmol/cm^3^ tissue
**VEGF_164_ binding to NRP-1**					
k_on_	3.13×10^6^	M^−1^s^−1^	2.77×10^−2^	6.41×10^−3^	(pmol/cm^3^ tissue)^−1^s^−1^
k_off_	1.00×10^−3^	s^−1^			
K_d_	3.12×10^2^	pM	3.52×10^−2^	1.52×10^−1^	pmol/cm^3^ tissue
**VEGF_164_ binding to GAGs**					
k_on_	4.20×10^5^	M^−1^s^−1^	3.72×10^−3^	-	(pmol/cm^3^ tissue)^−1^s^−1^
k_off_	1.00×10^−2^	s^−1^			
K_d_	2.40×10^1^	nM	2.71	-	pmol/cm^3^ tissue
**Coupling of NRP-1 and VEGFR-2**					
k_c V164R2,N1_	3.10×10^13^	(mol/cm^2^)^−1^s^−1^	1.99×10^−1^	1.59×10^−2^	(pmol/cm^3^ tissue)^−1^s^−1^
k_off V164R2,N1_	1.00×10^−3^	s^−1^			
k_c V164N1,R2_	1.00×10^14^	(mol/cm^2^)^−1^s^−1^	6.41×10^−1^	5.13×10^−2^	(pmol/cm^3^ tissue)^−1^s^−1^
k_off V164N1,R2_	1.00×10^−3^	s^−1^			
**Coupling of NRP-1 and VEGFR-1**					
k_c R1,N1_	1.00×10^14^	(mol/cm^2^)^−1^s^−1^	6.41×10^−1^	5.13×10^−2^	(pmol/cm^3^ tissue)^−1^s^−1^
k_dissoc R1,N1_	1.00×10^−2^	s^−1^			
**VEGFR internalization**					
k_int, R_	2.80×10^−4^	s^−1^			
k_int, C_	2.80×10^−4^	s^−1^			
**VEGF binding to anti-VEGF** *					
k_on_	1.13×10^8^	M^−1^s^−1^	1.00	2.32×10^−1^	(pmol/cm^3^ tissue)^−1^s^−1^
k_off_	4.23×10^−5^	s^−1^			
K_d_	0.37	pM	4.18×10^−5^	1.81×10^−4^	pmol/cm^3^ tissue

*Optimized parameter values.

Conversions:

Tissue: 1.13×10^8^ (pmol/cm^3^ tissue)/M and 1.56×10^14^ (pmol/cm^3^ tissue)/(mol/cm^2^ EC).

Blood: 4.88×10^8^ (pmol/cm^3^ tissue)/M and 1.95×10^15^ (pmol/cm^3^ tissue)/(mol/cm^2^ EC).

## Results

**Figure 3:**
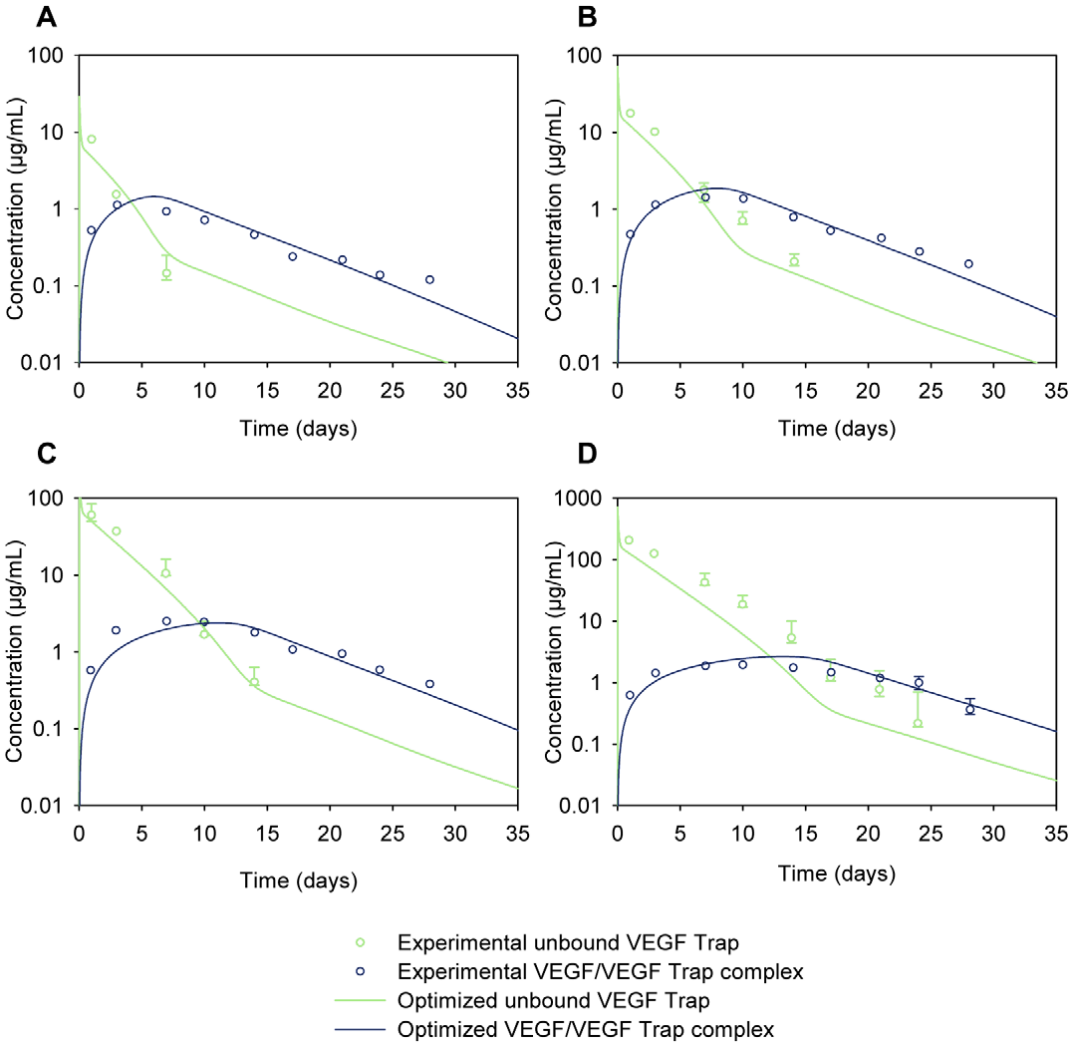
Parameter optimization. Model parameters were optimized computationally to fit simulation results (solid lines) to experimental data (open circles) [16] for the concentration profiles of the unbound VEGF Trap and the VEGF/VEGF Trap complex. VEGF Trap was injected intravenously (into the blood compartment) at (A) 1 mg/kg, (B) 2.5 mg/kg, (C) 10 mg/kg, and (D) 25 mg/kg.

The experiments of Rudge *et al.* detail the effects of injecting VEGF Trap subcutaneously into mice [16]. The bioavailability of VEGF Trap and the VEGF/VEGF Trap complex is the same whether the drug is injected subcutaneously or intravenously [16]. Since our model explicitly includes the blood compartment, it is straightforward to model drug delivery directly into the blood. Thus, we have simulated an intravenous injection of the anti-VEGF agent. The experimental plasma concentration profiles of the VEGF/VEGF Trap complex and unbound VEGF Trap in the mice at different doses of VEGF Trap are shown in Figure 3. We have used these data to develop an experiment-based model.

### Free parameters

In order to fit the simulation results to the experimental concentration profiles, the values of a subset of model parameters were chosen to be optimized, specifically the following five parameters: VEGF secretion rate, lymphatic drainage rate, clearance rate of VEGF Trap, clearance rate of the VEGF/VEGF Trap complex, and the dissociation constant of VEGF and VEGF Trap. These five parameters are denoted as the free parameters. We chose to optimize these free parameters based on the uncertainty of the parameter values in the literature and our sensitivity analysis. The sensitivity analysis systematically investigated the impact of individual parameters on the model output (namely, the concentration profiles of VEGF, VEGF Trap, and the complex; data not shown).

The multiple VEGF isoforms have different expression levels depending on the species and tissue type [59]. In mouse skeletal muscle, the mRNA expression ratio of VEGF_164_:VEGF_120_ was measured to be 92%:8% [60]. In our model, we assume that the secretion rates of VEGF_120_ and VEGF_164_ by the myocytes follow this ratio as well. In the parameter estimation, the lower and upper bounds that the secretion rate of VEGF_164_ (*q_V164_*) was allowed to take were 0.01 and 0.20 molecules/cell/s, respectively. The ratio between the secretion rate of VEGF_120_ (*q_V120_*) and that of VEGF_164_ (*q_V164_*) is taken to be 8∶92. The total VEGF secretion rate is the sum of *q_V64_* and *q_V120_*.

The lymphatic drainage rate has been measured to be 0.2–0.3 mL/hr in suckling rats weighing 35–45 g [61], corresponding to approximately 7×10^−5^ cm^3^/s. This is consistent with observations in male mice, where the lymph flow rate was measured to be 4 µL/min, corresponding to 0.24 mL/hr [62]. In the parameter estimation, the lower and upper bounds that the lymphatic drainage rate (*k_L_*) was allowed to take were 7.0×10^−6^ and 7.0×10^−4^ cm^3^/s, respectively.

The half-life of VEGF Trap in mouse serum was reported as 72 h [63], which corresponds to a clearance rate (*c_A_*) of 1.60×10^-4^ min^−1^. This clearance rate is a first estimate as we assume that the elimination of VEGF Trap *in vivo* is a first order process. In addition, the half-life in the serum is not necessarily the same as the half-life in the plasma because molecules contained in platelets may not be degraded at the same rate as molecules in direct contact with the plasma. Since the VEGF/VEGF-Trap complex can also be sequestered and transported by platelets, the same argument holds for its clearance rate (*c_VA_*). Hence, both clearances were chosen to be free parameters, and their allowable lower and upper bounds in the parameter estimation were both 1.60×10^−5^ and 1.60×10^−3^ min^−1^, respectively. This corresponds to a half-life of 4.33×10^4^ and 4.33×10^2^ min, respectively.

Kinetic parameters for the binding and unbinding of VEGF to the anti-VEGF agent are based on the pharmacokinetic parameters for VEGF Trap: The unbinding rate (*k_off_*) is 4.23×10^−5^ s^−1^ [64], [65]. The equilibrium dissociation constant (*K_d_*) has been reported to range from <1 pM [16] to 5 pM [65]. In the parameter estimation, the lower and upper bounds that the dissociation constant was allowed to take were 0.25 and 5 pM, respectively.

### Parameter estimation

The estimation of the free parameters was treated as a non-linear optimization problem. We used an automated optimization approach that explored the free parameter space and returned the combination of free parameter values that allowed the simulation results to best fit the experimental data from Rudge *et. al* [16] (Figure 3, open circles). The parameter optimization was performed using MATLAB®, which solves the non-linear least squares problem and uses the trust-region-reflective optimization algorithm [66], [67]. The objective function that was minimized was the weighted sum of squared residuals (WSSR) and obeyed:
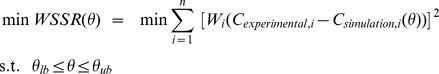



where *C_experimental,i_* is the *i^th^* experimentally measured plasma concentration data point of VEGF Trap or VEGF/VEGF Trap complex (Figure 3, open circles), and *C_simulation,i_*(*θ*) is the *i^th^* simulated plasma concentration at the corresponding time point (Figure 3, solid lines). The number of experimental measurements *n* includes both VEGF Trap and VEGF/VEGF Trap complex concentrations for all four doses of VEGF Trap, totaling 58 data points. The weights *W_i_* were taken to be 1/*C_experimental,i_* [68]. *θ_ lb_* and *θ_ ub_* are the lower and upper bounds, respectively, that the free parameters were allowed to take.

Because the optimization function only minimizes the objective function locally, twenty optimization trials were performed such that for each trial, the initial value of each of the free parameters was randomly generated within *θ_ lb_* and *θ_ ub_*. The final set of optimized free parameters was taken to be the trial that yielded the smallest WSSR. The final optimized free parameters were: *q_V164_*  =  0.0626 molecules/cell/s, *k_L_*  =  7.00×10^−6^ cm^3^/s, *c_A_*  =  8.86×10^−4^ min^−1^, *c_VA_*  =  2.79×10^−4^ min^−1^, and *K_d_*  =  0.37 pM. These optimized values yielded a WSSR value of 8.137. For comparison, the largest WSSR of the twenty trials was 8.382. The results of the optimization are summarized in Table 6. The optimized values from each of the twenty optimization trials are presented in Table S1. With the free parameters optimized, we completed the formulation of the model. This resulting model is denoted as the *optimized* model, and all subsequent results are based on this model. Using the optimized model, we were able to fit the concentration profiles for VEGF Trap and the VEGF/VEGF Trap complex (Figure 3, solid lines).

**Table 6:** Summary of parameter optimization (n = 20).

	Lower bound	Upper bound	Min	Max	Optimal*	Unit
VEGF_164_ secretion rate	0.01	0.20	0.0544	0.0647	0.0626	molecules/cell/s
Lymphatic drainage	7.00×10^−6^	7.00×10^−4^	7.00×10^−6^	2.73×10^−5^	7.00×10^−6^	cm^3^/s
Clearance of VEGF Trap	1.60×10^−5^	1.60×10^−3^	8.45×10^−4^	9.38×10^−4^	8.86×10^−4^	min^−1^
Clearance of VEGF/VEGF Trap complex	1.60×10^−5^	1.60×10^−3^	2.16×10^−4^	3.20×10^−4^	2.79×10^−4^	min^−1^
K_d_ of VEGF and VEGF Trap	0.25	5.00	0.32	0.48	0.37	pM

*Of the 20 trials, the optimal trial was the one that yielded the smallest weighted sum of squared residuals.

### Concentrations of molecular species

The optimized model was first simulated to achieve steady-state concentrations. The steady-state concentrations of VEGF were 0.098 pM and 5.27 pM in the blood and tissue, respectively. This plasma concentration is consistent with experimental measurements in mice. Multiple studies have shown that the VEGF plasma concentration is low in mice. It ranges from 13.7 pg/mL [69] to 65 pg/mL [14], which corresponds to 0.30 to 1.4 pM, respectively, converted using a VEGF molecular weight of 45 kDa. To our knowledge, the concentration of VEGF in the tissue interstitial space has not been measured experimentally.

**Figure 4:**
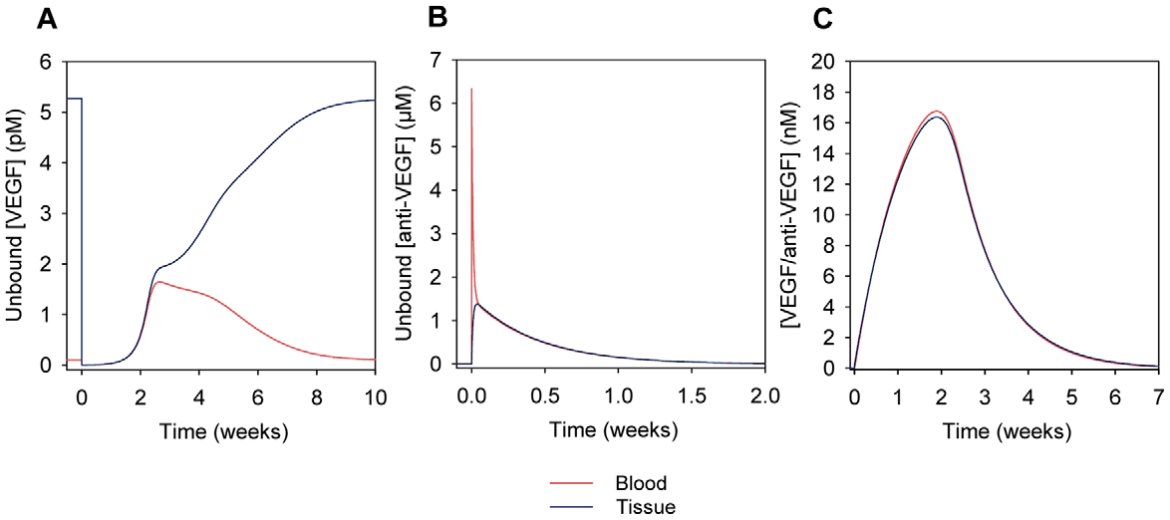
Concentration profiles following the injection of the anti-VEGF agent (VEGF Trap). A 25 mg/kg injection of anti-VEGF in the blood at time 0 was simulated using the optimized parameters. The profiles of (A) unbound VEGF, (B) unbound anti-VEGF, and (C) VEGF/anti-VEGF complex are shown in the blood (red) and tissue (blue) compartments. In (A), VEGF level in the tissue drops significantly after injection. Blood VEGF concentration increases to levels greater than steady-state reaching a maximum at 2.7 weeks post-injection, and returns to original steady-state levels by approximately 10 weeks after injection. Note that the axes of the panels are on different scales.

After the steady-state was achieved, we simulated an injection of 25 mg/kg VEGF Trap into the blood compartment, which lasted for one minute. The concentration of unbound VEGF in the blood and tissue compartments decreases after the injection as VEGF Trap and VEGF bind to form a complex (Figure 4A). At 2.7 weeks post-injection, the concentration of unbound VEGF in the blood reaches a maximum of 1.6 pM, which is greater than the initial pre-injection concentration. This increase in the plasma concentration of unbound VEGF was also observed in the human model [8] and has been hypothesized to be due to a “shuttling” effect where, on average, the VEGF Trap forms a complex with VEGF in the tissue compartment, after which the complex is transported into the blood where it dissociates and releases the free VEGF.

It is interesting to note that while the increase in blood VEGF levels occurs over the course of a few weeks in this mouse model, the human model predicts this similar increase over the course of only a few days. The difference in the time scale of the VEGF increase can be attributed to the fact that in the human model, the kinetic parameters of the anti-VEGF agent were based on those of bevacizumab (Avastin), which are different from those of VEGF Trap [8]. The dissociation constant of bevacizumab and VEGF is greater than that of VEGF Trap and VEGF, which means that bevacizumab does not bind as strongly to VEGF as VEGF Trap does. The larger dissociation constant of bevacizumab allowed the concentrations of VEGF to return to pre-injection levels in the human model more rapidly whereas in this mouse model, the concentrations of unbound VEGF returned to the pre-injection levels only after 10 weeks post-injection.

Figure 4B shows that after a rapid increase in concentration following the injection, the concentration of unbound VEGF Trap decreases and is no longer in the body by 2 weeks after injection. In Figure 4C, the concentration of the VEGF/VEGF Trap complex reaches maximum levels of 16.8 and 16.4 nM in the blood and tissue, respectively, at 1.9 weeks post-injection. The complex is cleared from the body by 7 weeks post-injection.

### Effect of VEGF secretion, clearance, and permeability on steady-state VEGF levels

**Figure 5:**
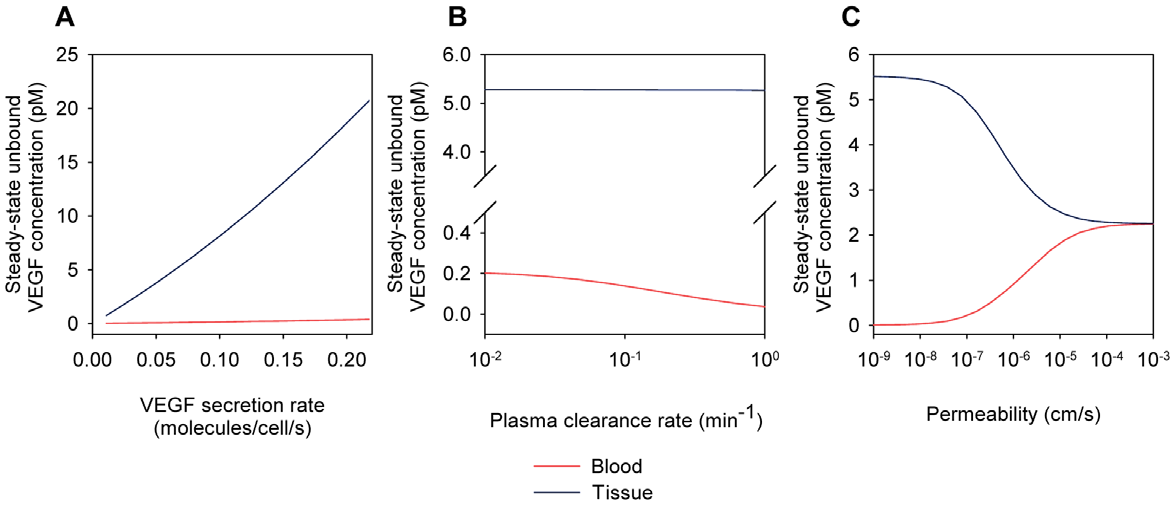
Steady-state concentration of free VEGF. (A) The steady-state concentration of VEGF in the tissue compartment but not in the blood is highly dependent on the VEGF secretion rate. (B) The VEGF concentration in the blood is more sensitive to the VEGF plasma clearance rate than the VEGF concentration in the tissue. (C) As microvascular permeability of VEGF increases, VEGF concentrations in the tissue and blood compartments equilibrate to 2.25 pM. For all simulations, unless the parameter is varied: VEGF_164_ secretion rate *q_V164_*  =  0.063 molecules/cell/s (optimized value), plasma clearance rate *c_V_*  =  0.23 min^−1^, and permeability *k_p_*  =  4.0×10^−8^ cm/s.

While the parameter optimization resulted in the optimized model, single parameter values can be varied individually to explore the robustness of the model output with respect to these individual parameters. To investigate the sensitivity of VEGF levels to systemic model parameters, VEGF secretion rate, VEGF clearance rate, and microvascular permeability to VEGF were varied individually. VEGF_164_ secretion rate was varied from 0.01 to 0.20 molecules/cell/s (Figure 5A). A 92%:8% expression ratio of VEGF_164_:VEGF_120_ was used in all simulations. While the steady-state concentration of unbound VEGF in the blood remained relatively low (< 0.4 pM) and relatively constant, the concentration of unbound VEGF in the tissue increased significantly with the secretion rate, reaching nearly 21 pM when the secretion rate of VEGF_164_ was 0.20 molecules/cell/s. Increasing the plasma clearance rate caused the depletion of VEGF from the blood (Figure 5B). As microvascular permeability was increased, the concentrations of unbound VEGF in the blood and tissue compartments equilibrated to 2.25 pM (Figure 5C).

### Effect of geometric tissue parameters on steady-state VEGF levels

In addition to systemic model parameters, geometric parameters were varied to examine their effect on steady-state VEGF levels. In particular, the length of the myonuclear domain was changed from 8.56 µm to 20 µm, as the latter value is closer to the upper limit reported in the literature [43], [44]. In this context, a myocyte is equivalent to a single myonuclear domain; hence, increasing the myonuclear domain length from 8.56 µm to 20 µm increased the surface area from 1.83×10^−5^ cm^2^ to 4.28×10^−5^ cm^2^. However, since the fraction of tissue that is occupied by myocytes is fixed, an increase in the myonuclear domain length resulted in a decrease in the total number of myocytes per unit volume of tissue. Since the number of NRP-1dimers per myocyte has a fixed value of 35,000 dimers/myocyte, the total number of NRP-1 per unit volume of tissue decreased. Additionally, because the secretion rate of VEGF is expressed in units of molecules/myonuclear domain/s, increasing the myonuclear domain length to 20 µm required that the optimized secretion rate be scaled up to 0.15 molecules/myonuclear domain/s to preserve the total number of VEGF molecules secreted per unit volume of tissue. By doing this, the only effective change that occurred as a result of increasing the myonuclear domain length was a decrease in the total NRP-1 concentration on the myocytes per unit volume of tissue. In this case, the concentration of unbound VEGF in the blood and tissue compartments increased to 0.11 and 6.70 pM, respectively (compared to the original concentrations of 0.098 and 5.27 pM, respectively). Thus, selecting a myonuclear domain length at the upper limit of the reported values increased the concentration of VEGF in the blood and tissue compartments 1.1- and 1.3-fold, respectively.

### Flow of molecular species between compartments at steady state

**Figure 6:**
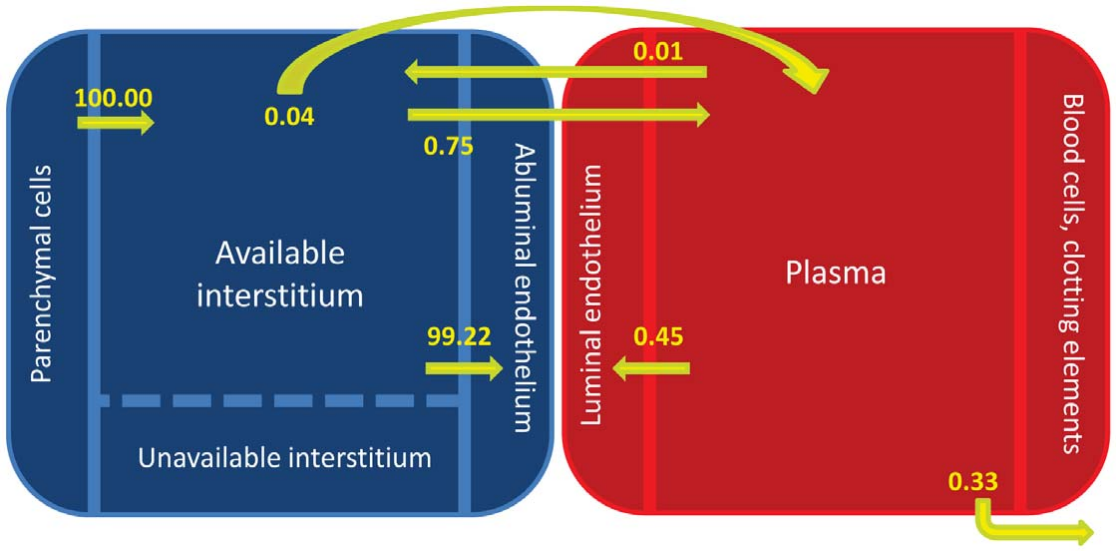
Normalized flows at steady-state. Flows of VEGF are normalized to that of the secretion flow. Most of the VEGF/receptor complexes are removed from the tissue through internalization. As with Figure 1, internalization in the tissue compartment includes both that of VEGF/receptor complexes on the abluminal surfaces of endothelial cells as well as VEGF bound to NRP-1 on myocyte cell surfaces. There is a net flow of VEGF from the tissue into the blood compartment via microvascular permeability.

The model predicted the flows of VEGF in the mouse at steady state (no injection of VEGF Trap) normalized to that of the secretion. The majority (99.2%) of VEGF produced by secretion was removed from the tissue compartment via internalization of the VEGF/receptor complexes (Figure 6). Specifically, 62.4% was removed via the internalization of VEGF/NRP-1 complexes on the myocytes, and 36.8% was removed via the internalization of VEGF/receptor complexes on the endothelial cells. Although the flows of VEGF from intravasation and extravasation were low compared to the secretion flow rate, there was a net flow of VEGF out of the tissue compartment into the blood compartment via microvascular permeability at steady state. Lymphatic drainage transported 0.04% of the secreted VEGF from the tissue to the blood. A small portion of the secreted VEGF was removed from the blood compartment via plasma clearance (0.33%).

### VEGF distribution in the body at steady state

**Figure 7:**
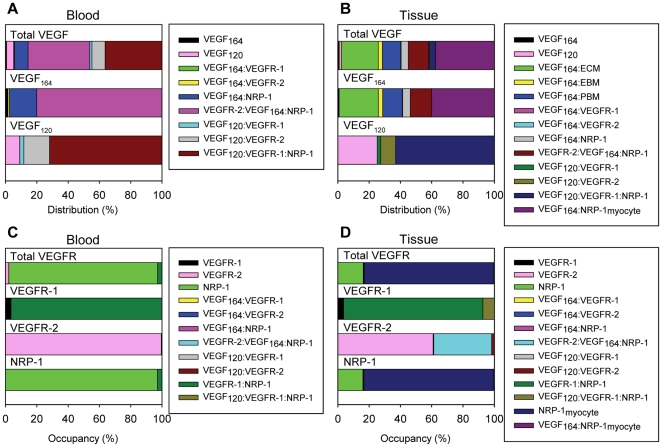
VEGF distributions and VEGFR occupancies at steady-state. The distributions of total VEGF and of the two individual isoforms are shown for the (A) blood and (B) tissue compartments. In the blood compartment, the majority of VEGF is found in the form of the VEGFR-2/VEGF_164_/NRP-1 and VEGF_120_/VEGFR-1/NRP-1 ternary complexes. In the tissue compartment, most of the VEGF is in the form of the VEGF_164_ isoform bound to NRP-1 on the myocytes. The occupancies of total VEGF receptors and of the individual receptors are shown for the (C) blood and (D) tissue compartments. Unbound NRP-1 on the luminal endothelial cell surface makes up the majority of total receptors and complexes in the blood compartment. Similarly, unbound NRP-1 on the abluminal endothelial cell and myocyte cell surfaces makes up the majority of total receptors and complexes in the tissue compartment.

The model provided an estimate of the steady-state fraction of total VEGF bound to receptors and to GAG chains located in the extracellular matrix and basement membranes. In the blood, 39.5% and 36.4% of total VEGF was in the form of the VEGFR-2/VEGF_164_/NRP-1 and VEGF_120_/VEGFR-1/NRP-1 ternary complexes, respectively (Figure 7A). Free circulating VEGF accounted for only 5.23% of the total VEGF in the blood. The ratio of unbound VEGF_164_ to unbound VEGF_120_ in the blood was 11.5%:88.5%. In the tissue, because of the large total surface area of myocytes compared to endothelial cells and thus the significantly larger number of total NRP-1 molecules on myocytes compared to endothelial cells, the majority of VEGF (37.6%) was bound to NRP-1 on the myocyte cell surfaces (Figure 7B). A significant portion of total VEGF (23.6%) was bound to the extracellular matrix. Unbound VEGF in the interstitial space comprised just 2.3% of the total VEGF in the tissue. The ratio of unbound VEGF_164_ to unbound VEGF_ 120_ in the tissue was 33.7%:66.3%. The effect of ratio between the secretion rate of VEGF_120_ and that of VEGF_164_ is clearly seen in the distribution of total VEGF, which closely resembles the distribution of VEGF_164_ (Figure 7B). This indicates that the majority of the total VEGF in the tissue is in the form of VEGF_164_, a direct result of the VEGF isoform secretion ratio VEGF_164_: VEGF_120_ being set to 92%:8%.

### VEGF receptor occupancy at steady state

Fractional occupancies of VEGF receptors at steady state were calculated. In the blood, unbound NRP-1 constituted 95.1% of the receptors on the luminal surface of endothelial cells (Figure 7C). Looking at VEGFR-1, VEGFR-2, and NRP-1 individually, we found that the majority of NRP-1 and VEGFR-2 remained unligated, and the majority of VEGFR-1 was coupled to NRP-1. This is because the concentration of unbound VEGF in the blood is small compared to the concentrations of these receptors. In the tissue, unligated NRP-1 on the myocytes and abluminal surfaces of the endothelial cells made up 82.8% and 16.0% of the total receptors, respectively, since there is more NRP-1 expressed per endothelial cell and per myocyte compared to VEGFR-1 and VEGFR-2 (Figure 7D). 89.0% of total VEGFR-1was found as VEGFR-1/ NRP-1. The majority of total VEGFR-2 in the tissue was found as either unbound VEGFR-2 (60.9%) or as VEGFR-2/VEGF_164_/NRP-1 (37.0%).

### Intercompartmental flows upon injection of VEGF Trap

**Figure 8:**
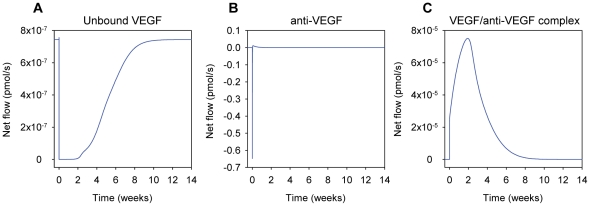
Intercompartmental flows following the intravenous injection of the anti-VEGF agent (VEGF Trap). Instantaneous net flow rates of (A) unbound VEGF, (B) anti-VEGF, and (C) VEGF/anti-VEGF complex are calculated upon a 25 mg/kg injection of anti-VEGF into the blood. A positive net flow indicates movement from the tissue into the blood via intravasation and lymphatics, and a negative net flow indicates movement from the blood into the tissue via extravasation. Note that the y-axes of the panels are on different scales.

Using the optimized model, we explored the effects of injecting VEGF Trap into the blood as a function of time, after an initial steady state had been achieved. To visualize the relative amounts of VEGF, VEGF Trap and VEGF/VEGF Trap complex moving between two compartments, the net flows of these molecular species after a 25 mg/kg injection of VEGF Trap into the blood were calculated. The net flow for each molecular species is the summation of the flows from intravasation, extravasation, and lymphatic drainage. The sign of the net flow indicates the net direction of flow of the molecule. Flow of a molecule from the tissue to the blood via intravasation and lymphatic drainage has a positive sign, while flow from the blood to the tissue via extravasation is considered to be negative. The flow of unbound VEGF remained positive during the course of the simulation and is smaller relative to the flow of the VEGF/VEGF Trap complex (Figure 8A). The model predicted that upon the intravenous injection of VEGF Trap, there is an initial flow of VEGF Trap from the blood to the tissue (Figure 8B). Then the direction of the flow is inverted (from tissue to blood) and deceases in intensity. The flow of the complex was positive during the entire time that it is in the body (Figure 8C). This suggests that quickly after the injection, VEGF Trap extravasates from the blood and binds to VEGF in the tissue. The complex then moves back into the blood via intravasation and lymphatics, after which it unbinds and releases VEGF in the blood, which is the proposed mechanism for the increase in blood VEGF levels after the injection and agrees with the shuttling effect predicted by Stefanini *et. al* [8]. Note that the flows for VEGF, VEGF Trap and the VEGF/VEGF Trap complex have different orders of magnitudes, as shown in Figure 8.

### VEGF distribution in the body upon injection of VEGF Trap

**Figure 9:**
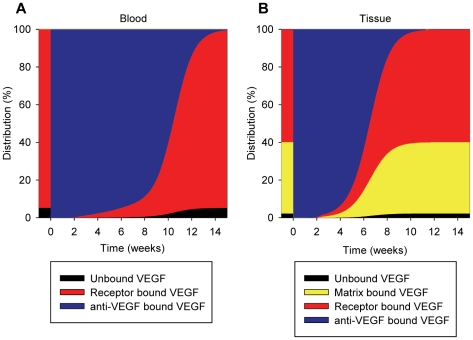
VEGF distributions upon injection of anti-VEGF (VEGF Trap). The distributions of VEGF in the (A) blood and (B) tissue compartments are shown subject to a 25 mg/kg injection of the anti-VEGF agent into the blood compartment at 0 weeks. Before the injection, 95% of VEGF in the blood compartment is receptor bound. In the tissue compartment, 38% of VEGF is sequestered in the extracellular matrix and endothelial and parenchymal basement membranes. 60% of VEGF is receptor-bound. Shortly following the injection of the anti-VEGF agent, essentially all of the VEGF in both compartments becomes sequestered by the anti-VEGF agent. By 14 weeks, VEGF distributions return to original steady-state levels.

The percentages of total VEGF in the two compartments were calculated after a 25 mg/kg intravenous injection of VEGF Trap. In the blood, most of VEGF became sequestered by VEGF Trap shortly after the injection (Figure 9A). A similar effect was seen in the tissue (Figure 9B). By 14 weeks, VEGF distributions in both compartments returned to the initial steady-state levels as in Figure 7A and 7B.

### VEGF receptor occupancy upon injection of VEGF Trap

**Figure 10:**
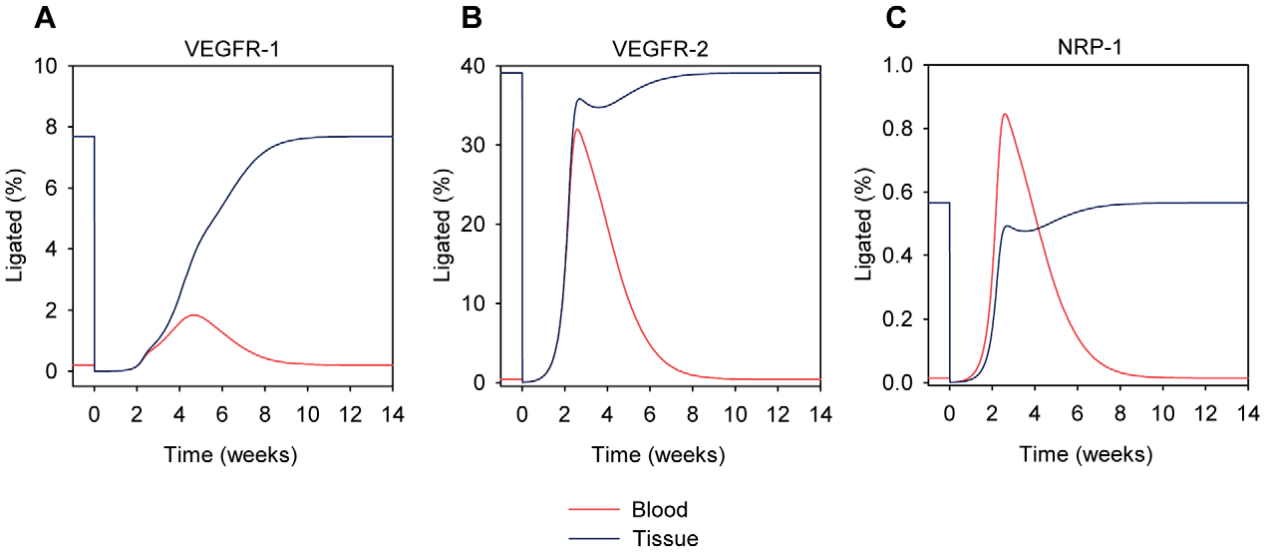
VEGF Receptor fractional occupancies upon injection of anti-VEGF (VEGF Trap). The fractional occupancies of (A) VEGFR-1, (B) VEGFR-2, and (C) NRP-1 are shown for the blood (red) and tissue (blue) compartments following a 25 mg/kg injection of the anti-VEGF agent into the blood compartment at 0 weeks. For all three receptors in the blood compartment, the percent of receptors ligated with VEGF decreases to essentially zero quickly after the injection of the anti-VEGF; however, the percent of ligated receptors then increases to values above pre-injection levels before returning to pre-injection levels. In the tissue compartment, this effect is not seen as the percent of ligated receptors decreases quickly after injection and then returns to pre-injection levels. Note that the y-axes of the panels are on different scales.

The receptor occupancies of the VEGF receptors were calculated following the injection of 25 mg/kg VEGF Trap into the blood compartment. The percent of ligated VEGFR-1 decreased in both tissues (Figure 10A). In the blood, the percent of ligated VEGFR-1 increased to levels above pre-injection before returning to pre-injection steady-state levels. The increase in ligated VEGFR-1 occurred because more VEGF is available in blood plasma, due to the shuttling effect described above. In the tissue, the percent of ligated VEGFR-1 simply returned to pre-injection levels. Similar behaviors were also seen for VEGFR-2 (Figure 10B), as well as for NRP-1 (Figure 10C) in both compartments.

### Predicted secretion rate of VEGF

The VEGF secretion rate from the muscle fibers was calculated by parameter optimization. From the twenty optimization trials that were performed, the minimum and maximum optimized VEGF_164_ secretion rates were 0.0544 and 0.0647 molecules/cell/s, respectively. In the trial that yielded the smallest WSSR, the VEGF_164_ secretion rate was 0.0626 molecules/cell/s, which was the value used in the optimized model. Since the expression ratio of VEGF_164_:VEGF_120_ is taken to be 92%:8% in accordance to Ee *et al* [61], the combined secretion rate of the two isoforms was then predicted to be 0.0680 molecules/cell/s, which is equivalent to 6.15×10^−9^ pmol/cm^2^/s, or 4.39×10^−6^ pmol/cm^3^ tissue/s, based on a myocyte cell surface area of 1.84×10^−5^ cm^2^/cell and a muscle fiber surface area of 713.68 cm^2^/cm^3^ tissue.

### Effect of VEGF degradation on parameter estimation

Up to this point, we have not included any specific degradation mechanisms of VEGF in the model. However, *in vivo*, circulating and tissular enzymes such as plasmin and matrix metalloproteinases actively degrade the VEGF ligand. Therefore, we have added a degradation term for VEGF in the normal tissue. Based on the half-life of human VEGF determined in various *in vitro* experiments [70]–[72], we have set the VEGF degradation rate constant to be 1.93×10^−4^ s^−1^ (1.16×10^−2^ min^−1^), which corresponds to a half-life of 60 minutes. We have assumed that in the blood, the clearance rate of VEGF includes degradation since the clearance rate constant is 20 times larger than that for degradation. With the addition of VEGF degradation in the normal tissue, an additional twenty optimization trials were performed to determine the optimal free parameter values as shown in Table S2. As before, the final set of optimized free parameters was taken to be the trial that yielded the smallest WSSR. The final optimized free parameters were: *q_V164_*  =  0.0627 molecules/cell/s, *k_L_*  =  7.00×10^−6^ cm^3^/s, *c_A_*  =  8.85×10^−4^ min^−1^, *c_VA_*  =  2.79×10^−4^ min^−1^, and *K_d_*  =  0.37 pM. These optimized values yielded a WSSR value of 8.161. The percent changes of the each of the free parameter values with and without degradation were all less than 1%, which suggests that the inclusion of VEGF degradation does not greatly affect the results obtained from the model when degradation was absent.

### Effect of VEGF degradation on steady-state VEGF levels

**Figure 11:**
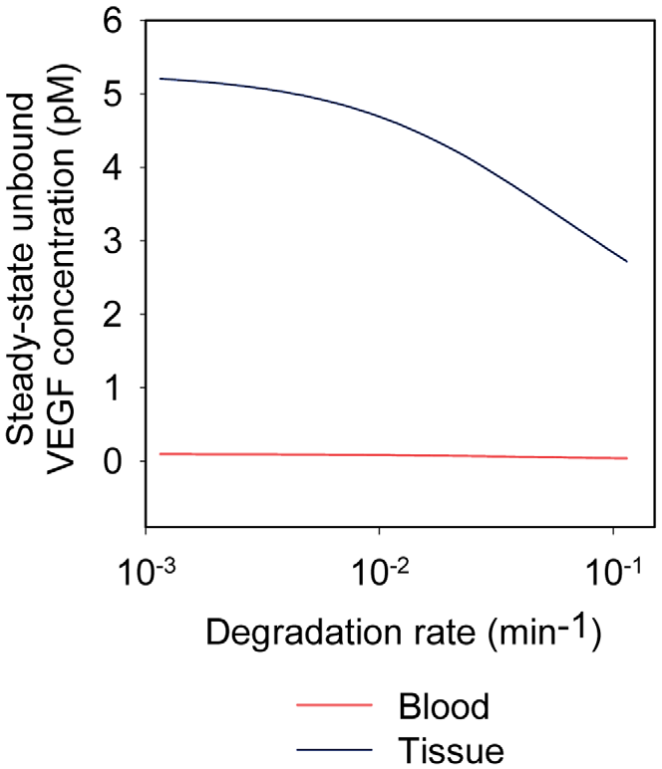
Effect of VEGF degradation on unbound VEGF levels. A VEGF degradation rate constant of 1.16×10^−2^ min^−1^ (corresponding to half-life of 60 minutes) in the normal tissue was added before re-performing the estimation of the free parameters. Using the new set of optimized parameter values, the steady-state concentrations of unbound VEGF were calculated as the degradation rate was varied. The concentration of unbound VEGF in the normal tissue decreases as the degradation rate increases.

Steady-state concentrations of unbound VEGF were simulated using the optimal free parameter values obtained with the inclusion of VEGF degradation. The steady-state concentrations of VEGF in the normal tissue and blood compartments were 4.61 pM and 0.082 pM, respectively. These constitute a 12% and 16% decrease in the steady-state concentrations of VEGF in the normal tissue and blood compartments, respectively, from the optimized model obtained before the addition of VEGF degradation. Additionally, the steady-state VEGF concentrations were determined as the VEGF degradation rate was varied (Figure 11). Unbound VEGF levels in the normal tissue and blood decrease as the VEGF degradation rate increases.

## Discussion

We have extended the previously developed compartmental model of VEGF distribution in humans to investigate the VEGF distribution in the mouse. As with the previous version of the human model [19], VEGF receptors are expressed on both abluminal and luminal surfaces of endothelial cells. NRP-1 is known to be expressed on muscle fibers; hence, one major change that was introduced in this model was the addition of NRP-1 on the myocyte cell surfaces to better reflect physiological conditions.

The effects of VEGF Trap on the distribution of VEGF in mice have been measured experimentally by Rudge *et al.* VEGF Trap was tested under physiological conditions (i.e. no tumor), which allowed us to fit our physiologically-based model of the mouse to the experimental data and to validate the model. In the previous model iterations, validating the model against experimental data involved adjusting the VEGF secretion rate until the concentration of VEGF in the blood was within the range reported in the literature. With the mouse model, five free parameters were estimated simultaneously, thus estimating ranges for several parameters that have high degrees of uncertainty. The parameter estimation was performed on a subset of the model parameters, selected based on how they influenced the model output.

Of these free parameters, the endogenous VEGF secretion rate by the myocytes and its predicted value was of particular interest, as direct experimental measurement of this parameter is non-trivial, and it is predicted to have a drastic effect on the concentration of unbound VEGF in the tissue. VEGF levels are typically measured from blood samples experimentally; however, different transport routes aside from VEGF secretion such as lymphatic drainage, microvascular permeability, internalization, and plasma clearance all contribute to the VEGF concentration measured in the blood. By allowing the VEGF secretion rate to be one of the free parameters, we were able to find the best fitted value for the secretion rate of VEGF by the myocytes, which is not significantly changed by the inclusion of VEGF degradation in the normal tissue.

Several assumptions used in the model contribute to model limitations. The different mouse tissues were represented by a spatially averaged compartment denoted as the tissue compartment. Skeletal muscle is the primary tissue for which the VEGF system is well characterized. Additionally, skeletal muscle constitutes a large percentage of the mouse body weight. Therefore, the majority of the properties of the tissue were based on available literature characterization of the mouse gastrocnemius muscle. To more accurately describe the mouse, specialized compartments may be added to better detail the distribution and flows of VEGF throughout the animal body. For example, it may be beneficial to distinguish a compartment representing the liver or the kidneys, as these organs play a role in the clearance of VEGF and may affect the estimated model parameters. Although vascular elements, such as pericytes, are also important in angiogenesis, for simplicity we have represented the vasculature using only endothelial cells. Including additional compartments and vascular structures would require experimental data for VEGF secretion and the density of VEGF receptors and co-receptors. We can easily expand the model as these data become available. Similarly, we could extend the model to include additional receptors and co-receptors, such as soluble VEGFR-1 or neuropilin-2, which also influence angiogenesis [73], [74]. These additional model elements can be added in a step-by-step manner in order to understand the effect that each has on the distribution of VEGF.

VEGF_120_ and VEGF_164_ are the two VEGF-A isoforms that are predominantly expressed and hence are the two isoforms included in our model. A human VEGF isoform, VEGF_189_ has been shown to correlate with xenotransplantability of colon cancer tumor in mice, where increased expression of this isoform was correlated with successful transplantations [75]. A similar correlation was observed in esophageal cancer transplantability [76]. Another experiment has shown that VEGF_189_ is expressed more predominantly in solid tumor xenografts than in primary tumors [2]. Adding the VEGF_189_/VEGF_188_ isoform in the mouse model may be important if the model is extended to include a tumor xenograft compartment to study cancer.

Degranulation of platelets have been shown to be a source of growth factors, such as VEGF, in the blood [77]. As more data become available, circulating platelets may be added to the model.

The *in silico* mouse model described here can be used to provide ranges for important biological parameters that are not easily measureable experimentally. Additionally, the model can serve as a foundation for exploring diseases dependent on angiogenesis and as a tool for predicting the effects of pro- and anti-angiogenic therapies. As mice are often used in preclinical settings to study diseases such as peripheral arterial disease, coronary artery disease, and cancer, the model also provides a computational framework to investigate these diseases in parallel with experimental studies.

## Supporting Information

Text S1 **Chemical reactions, system of ordinary differential equations describing the model, and glossary.**
(PDF)

Table S1 **Optimized parameter values from twenty optimization trials.**
(DOC)

Table S2 **Optimized parameter values from twenty optimization trials when VEGF degradation is included.**
(DOC)
