# Supplementary material for: A Two-Compartment Model of VEGF Distribution in the Mouse
Source: PLoS One. 2011 Nov 8;6(11):e27514. doi: 10.1371/journal.pone.0027514 (PMC3210788; doi:10.1371/journal.pone.0027514)
Supplement: Text S1 — Chemical reactions, system of ordinary differential equations describing the model, and glossary. (PDF) [file pone.0027514.s001.pdf]

Text S1

Chemical reactions

The relevant chemical reactions are presented here (molecular species and parameters are defined in the glossary):

Tissue

$$V_{164} + M_{ECM} \underset{k_{off,V164MECM}}{\overset{k_{on,V164,MECM}}{\rightleftharpoons}} V_{164}M_{ECM}$$

$$V_{164} + M_{EBM} \underset{k_{off,V164MEBM}}{\overset{k_{on,V164,MEBM}}{\rightleftharpoons}} V_{164}M_{EBM}$$

$$V_{164} + M_{PBM} \underset{k_{off,V164MPBM}}{\overset{k_{on,V164,MPBM}}{\rightleftharpoons}} V_{164}M_{PBM}$$

$$V_{164} + N_{1,myo} \underset{k^{myo}_{off,V164N1}}{\overset{k^{myo}_{on,V164,N1}}{\rightleftharpoons}} V_{164}N_{1,myo}$$

Tissue and blood

$$V_{164} + R_1 \underset{k_{off,V164R1}}{\overset{k_{on,V164,R1}}{\rightleftharpoons}} V_{164}R_1$$

$$V_{164} + R_2 \underset{k_{off,V164R2}}{\overset{k_{on,V164,R2}}{\rightleftharpoons}} V_{164}R_2$$

$$V_{164} + N_1 \underset{k_{off,V164N1}}{\overset{k_{on,V164,N1}}{\rightleftharpoons}} V_{164}N_1$$

$$V_{164}N_1 + R_2 \underset{k_{off,V164N1R2}}{\overset{k_{c,V164N1,R2}}{\rightleftharpoons}} R_2V_{164}N_1$$

$$V_{164}R_2 + N_1 \underset{k_{off,V164R2N1}}{\overset{k_{c,V164R2,N1}}{\rightleftharpoons}} R_2V_{164}N_1$$

$$V_{120} + R_1 \underset{k_{off,V120R1}}{\overset{k_{on,V120,R1}}{\rightleftharpoons}} V_{120}R_1$$

$$V_{120} + R_2 \underset{k_{off,V120R2}}{\overset{k_{on,V120,R2}}{\rightleftharpoons}} V_{120}R_2$$

$$V_{120} + R_1N_1 \underset{k_{off,V120R1}}{\overset{k_{on,V120,R1}}{\rightleftharpoons}} V_{120}R_1N_1$$

$$V_{120}R_1 + N_1 \underset{k_{dissoc,R1N1}}{\overset{k_{c,R1,N1}}{\rightleftharpoons}} V_{120}R_1N_1$$

$$R_1 + N_1 \underset{k_{dissoc,R1N1}}{\overset{k_{c,R1,N1}}{\rightleftharpoons}} R_1N_1$$

$$V_{164} + A \underset{k_{off,V164A}}{\overset{k_{on,V164,A}}{\rightleftharpoons}} V_{164}A$$

$$V_{120} + A \underset{k_{off,V120A}}{\overset{k_{on,V120,A}}{\rightleftharpoons}} V_{120}A$$

Equations

The complete list of ordinary differential equations is presented below:

$$\begin{aligned}
\frac{d[V_{164}]_N}{dt} = {} & q_{V164}^N - k_{on,V164,MEBM}^N [V_{164}]_N [M_{EBM}]_N + k_{off,V164,MEBM}^N [V_{164}M_{EBM}]_N \\
& - k_{on,V164,MPBM}^N [V_{164}]_N [M_{PBM}]_N + k_{off,V164,MPBM}^N [V_{164}M_{PBM}]_N \\
& - k_{on,V164,MECM}^N [V_{164}]_N [M_{ECM}]_N + k_{off,V164,MECM}^N [V_{164}M_{ECM}]_N \\
& - k_{on,V164,R1}^N [V_{164}]_N [R_1]_N + k_{off,V164R1}^N [V_{164}R_1]_N \\
& - k_{on,V164,R2}^N [V_{164}]_N [R_2]_N + k_{off,V164R2}^N [V_{164}R_2]_N \\
& - k_{on,V164,N1}^N [V_{164}]_N [N_1]_N + k_{off,V164N1}^N [V_{164}N_1]_N \\
& - k_{on,V164,N1}^{N,myo} [V_{164}]_N [N_1]_{N,myo} + k_{off,V164N1}^{N,myo} [V_{164}N_1]_{N,myo} \\
& - k_{on,V164,A}^N [V_{164}]_N [A]_N + k_{off,V164A}^N [V_{164}A]_N \\
& - \left(\frac{k_L + k_{pV}^{NB} S_{NB}}{U_N} \right) \frac{[V_{164}]_N}{K_{AV,N}} + k_{pV}^{BN} \frac{S_{NB}}{U_N} \frac{U_B}{U_P} [V_{164}]_B
\end{aligned} \tag{S.1}$$

$$\begin{aligned}
\frac{d[V_{120}]_N}{dt} = {} & q_{V120}^N - k_{on,V120,R1}^N [V_{120}]_N [R_1]_N + k_{off,V120R1}^N [V_{120}R_1]_N \\
& - k_{on,V120,R1N1}^N [V_{120}]_N [R_1N_1]_N + k_{off,V120R1N1}^N [V_{120}R_1N_1]_N \\
& - k_{on,V120,R2}^N [V_{120}]_N [R_2]_N + k_{off,V120R2}^N [V_{120}R_2]_N \\
& - k_{on,V120,A}^N [V_{120}]_N [A]_N + k_{off,V120A}^N [V_{120}A]_N \\
& - \left(\frac{k_L + k_{pV}^{NB} S_{NB}}{U_N} \right) \frac{[V_{120}]_N}{K_{AV,N}} + k_{pV}^{BN} \frac{S_{NB}}{U_N} \frac{U_B}{U_P} [V_{120}]_B
\end{aligned} \tag{S.2}$$

$$\begin{aligned}
\frac{d[V_{164}]_B}{dt} = {} & -c_{V164} [V_{164}]_B - k_{on,V164,R1}^B [V_{164}]_B [R_1]_B + k_{off,V164R1}^B [V_{164}R_1]_B \\
& - k_{on,V164,R2}^B [V_{164}]_B [R_2]_B + k_{off,V164R2}^B [V_{164}R_2]_B \\
& - k_{on,V164,N1}^B [V_{164}]_B [N_1]_B + k_{off,V164N1}^B [V_{164}N_1]_B \\
& - k_{on,V164,A}^B [V_{164}]_B [A]_B + k_{off,V164A}^B [V_{164}A]_B \\
& - \frac{k_{pV}^{BN} S_{NB}}{U_p} [V_{164}]_B + \left(\frac{k_L + k_{pV}^{NB} S_{NB}}{U_B} \right) \frac{[V_{164}]_N}{K_{AV,N}}
\end{aligned} \tag{S.3}$$

$$\frac{d[V_{120}]_B}{dt} = -c_{V120}[V_{120}]_B - k^B_{on,V120,R1}[V_{120}]_B[R_1]_B + k^B_{off,V120R1}[V_{120}R_1]_B$$
$$-k^B_{on,V120,R1N1}[V_{120}]_B[R_1N_1]_B + k^B_{off,V120R1N1}[V_{120}R_1N_1]_B$$
$$-k^B_{on,V120,R2}[V_{120}]_B[R_2]_B + k^B_{off,V120R2}[V_{120}R_2]_B \quad \text{(S.4)}$$
$$-k^B_{on,V120,A}[V_{120}]_B[A]_B + k^B_{off,V120A}[V_{120}A]_B$$
$$-\frac{k^{BN}_{pV}S_{NB}}{U_p}[V_{120}]_B + \left(\frac{k_L + k^{NB}_{pV}S_{NB}}{U_B}\right)\frac{[V_{120}]_N}{K_{AV,N}}$$

$$\frac{d[M_{EBM}]_N}{dt} = -k^N_{on,V164,MEBM}[V_{164}]_N[M_{EBM}]_N + k^N_{off,V164MEBM}[V_{164}M_{EBM}]_N \quad \text{(S.5)}$$

$$\frac{d[M_{PBM}]_N}{dt} = -k^N_{on,V164,MPBM}[V_{164}]_N[M_{PBM}]_N + k^N_{off,V164MPBM}[V_{164}M_{PBM}]_N \quad \text{(S.6)}$$

$$\frac{d[M_{ECM}]_N}{dt} = -k^N_{on,V164,MECM}[V_{164}]_N[M_{ECM}]_N + k^N_{off,V164MECM}[V_{164}M_{ECM}]_N \quad \text{(S.7)}$$

$$\frac{d[V_{164}M_{EBM}]_N}{dt} = k^N_{on,V164,MEBM}[V_{164}]_N[M_{EBM}]_N - k^N_{off,V164MEBM}[V_{164}M_{EBM}]_N \quad \text{(S.8)}$$

$$\frac{d[V_{164}M_{PBM}]_N}{dt} = k^N_{on,V164,MPBM}[V_{164}]_N[M_{PBM}]_N - k^N_{off,V164MPBM}[V_{164}M_{PBM}]_N \quad \text{(S.9)}$$

$$\frac{d[V_{164}M_{ECM}]_N}{dt} = k^N_{on,V164,MECM}[V_{164}]_N[M_{ECM}]_N - k^N_{off,V164MECM}[V_{164}M_{ECM}]_N \quad \text{(S.10)}$$

$$\frac{d[R_1]_N}{dt} = s^N_{R1} - k^N_{int,R1}[R_1]_N - k^N_{on,V164,R1}[V_{164}]_N[R_1]_N + k^N_{off,V164R1}[V_{164}R_1]_N$$
$$-k^N_{on,V120,R1}[V_{120}]_N[R_1]_N + k^N_{off,V120R1}[V_{120}R_1]_N \quad \text{(S.11)}$$
$$-k^N_{c,R1,N1}[N_1]_N[R_1]_N + k^N_{dissoc,R1N1}[R_1N_1]_N$$

$$\frac{d[R_2]_N}{dt} = s^N_{R2} - k^N_{int,R2}[R_2]_N - k^N_{on,V120,R2}[V_{120}]_N[R_2]_N + k^N_{off,V120R2}[V_{120}R_2]_N$$
$$-k^N_{on,V164,R2}[V_{164}]_N[R_2]_N + k^N_{off,V164R2}[V_{164}R_2]_N \quad \text{(S.12)}$$
$$-k^N_{c,V164N1,R2}[V_{164}N_1]_N[R_2]_N + k^N_{off,V164N1,R2}[R_2V_{164}N_1]_N$$

$$\begin{aligned}\frac{d[N_1]_N}{dt} &= s_{N1}^N - k_{int,N1}^N[N_1]_N - k_{c,V120R1,N1}^N[V_{120}R_1]_N[N_1]_N + k_{dissoc,R1N1}^N[V_{120}R_1N_1]_N \\ &-k_{c,R1,N1}^N[N_1]_N[R_1]_N + k_{dissoc,R1N1}^N[R_1N_1]_N \\ &-k_{on,V164,N1}^N[V_{164}]_N[N_1]_N + k_{off,V164N1}^N[V_{164}N_1]_N \\ &-k_{c,V164R2,N1}^N[V_{164}R_2]_N[N_1]_N + k_{off,V164R2,N1}^N[R_2V_{164}N_1]_N\end{aligned} \quad (S.13)$$

$$\begin{aligned}\frac{d[R_1]_B}{dt} &= s_{R1}^B - k_{int,R1}^B[R_1]_B - k_{on,V164,R1}^B\ [V_{164}]_B[R_1]_B + k_{off,V164R1}^B[V_{164}R_1]_B \\ &-k_{on,V120,R1}^B[V_{120}]_B[R_1]_B + k_{off,V120R1}^B[V_{120}R_1]_B \\ &-k_{c,R1,N1}^B[N_1]_B[R_1]_B + k_{dissoc,R1N1}^B[R_NN_1]_B\end{aligned} \quad (S.14)$$

$$\begin{aligned}\frac{d[R_2]_B}{dt} &= s_{R2}^B - k_{int,R2}^B\ [R_2]_B - k_{on,V120,R2}^B[V_{120}]_B[R_2]_B + k_{off,V120R2}^B[V_{120}R_2]_B \\ &-k_{on,V164,R2}^B[V_{164}]_B[R_2]_B + k_{off,V164R2}^B[V_{164}R_2]_B \\ &-k_{c,V164N1,R2}^B[V_{164}N_1]_B[R_2]_B + k_{off,V164N1,R2}^B[R_2V_{164}N_1]_B\end{aligned} \quad (S.15)$$

$$\begin{aligned}\frac{d[N_1]_B}{dt} &= s_{N1}^B - k_{int,N1}^B[N_1]_B - k_{c,V120R1,N1}^B[V_{120}R_1]_B[N_1]_B + k_{dissoc,R1N1}^B[V_{120}R_1N_1]_B \\ &-k_{c,R1,N1}^B[N_1]_B[R_1]_B + k_{dissoc,R1N1}^B[R_1N_1]_B \\ &-k_{on,V164,N1}^B[V_{164}]_B[N_1]_B + k_{off,V164N1}^B[V_{164}N_1]_B \\ &-k_{c,V164R2,N1}^B[V_{164}R_2]_B[N_1]_B + k_{off,V164R2,N1}^B[R_2V_{164}N_1]_B\end{aligned} \quad (S.16)$$

$$\frac{d[V_{164}R_1]_N}{dt} = -k_{int,V164R1}^N[V_{164}R_1]_N + k_{on,V164,R1}^N[V_{164}]_N[R_1]_N - k_{off,V164R1}^N[V_{164}R_1]_N \quad (S.17)$$

$$\begin{aligned}\frac{d[V_{164}R_2]_N}{dt} &= -k_{int,V164R2}^N[V_{164}R_2]_N + k_{on,V164,R2}^N[V_{164}]_N[R_2]_N - k_{off,V164R2}^N[V_{164}R_2]_N \\ &-k_{c,V164R2,N1}^N[V_{164}R_2]_N[N_1]_N + k_{off,V164R2N1}^N[R_2V_{164}N_1]_N\end{aligned} \quad (S.18)$$

$$\begin{aligned}\frac{d[V_{164}N_1]_N}{dt} &= -k_{int,V164N1}^N[V_{164}N_1]_N + k_{on,V164,N1}^N[V_{164}]_N[N_1]_N - k_{off,V164N1}^N[V_{164}N_1]_N \\ &-k_{c,V164N1,R2}^N[V_{164}N_1]_N[R_2]_N + k_{off,V164N1R2}^N[R_2V_{164}N_1]_N\end{aligned} \quad (S.19)$$

$$\begin{aligned}\frac{d[R_2V_{164}N_1]_N}{dt} &= -k_{int,V164R2N1}^N[R_2V_{164}N_1]_N \\ &+k_{c,V164R2,N1}^N[V_{164}R_2]_N[N_1]_N - k_{off,V164R2N1}^N[R_2V_{164}N_1]_N \\ &+k_{c,V164N1,R2}^N[V_{164}N_1]_N[R_2]_N - k_{off,V164N1R2}^N[R_2V_{164}N_1]_N\end{aligned} \quad (S.20)$$

$$\frac{d[V_{120}R_1]_N}{dt} = -k^N_{int,V120R1}[V_{120}R_1]_N$$
$$+k^N_{on,V120,R1}[V_{120}]_N[R_1]_N - k^N_{off,V120R1}[V_{120}R_1]_N \quad \text{(S.21)}$$
$$-k^N_{c,R1,N1}[V_{120}R_1]_N[N_1]_N + k^N_{dissoc,R1N1}[V_{120}R_1N_1]_N$$

$$\frac{d[V_{120}R_2]_N}{dt} = -k^N_{int,V120R2}[V_{120}R_2]_N + k^N_{on,V120,R2}[V_{120}]_N[R_2]_N - k^N_{off,V120R2}[V_{120}R_2]_N \quad \text{(S.22)}$$

$$\frac{d[R_1N_1]_N}{dt} = -k^N_{int,R1N1}[R_1N_1]_N$$
$$+k^N_{c,R1,N1}[R_1]_N[N_1]_N - k^N_{dissoc,R1N1}[R_1N_1]_N \quad \text{(S.23)}$$
$$-k^N_{on,V120,R1}[V_{120}]_N[R_1N_1]_N + k^N_{off,V120R1}[V_{120}R_1N_1]_N$$

$$\frac{d[V_{120}R_1N_1]_N}{dt} = -k^N_{intV120R1N1}[V_{120}R_1N_1]_N$$
$$+k^N_{c,V120R1,N1}[V_{120}R_1]_N[N_1]_N - k^N_{dissoc,V120N1}[V_{120}R_1N_1]_N \quad \text{(S.24)}$$
$$+k^N_{on,V120R1N1}[V_{120}]_N[R_1N_1]_N - k^N_{off,V120R1N1}[V_{120}R_1N_1]_N$$

$$\frac{d[V_{164}R_1]_B}{dt} = -k^B_{int,V164R1}[V_{164}R_1]_B + k^B_{on,V164,R1}[V_{164}]_B[R_1]_B - k^B_{off,V164R1}[V_{164}R_1]_B \quad \text{(S.25)}$$

$$\frac{d[V_{164}R_2]_B}{dt} = -k^B_{int,V164R2}[V_{164}R_2]_B + k^B_{on,V164,R2}[V_{164}]_B[R_2]_B - k^B_{off,V164R2}[V_{164}R_2]_B \quad \text{(S.26)}$$

$$\frac{d[V_{164}N_1]_B}{dt} = -k^B_{int,V164N1}[V_{164}N_1]_B + k^B_{on,V164,N1}[V_{164}]_B[N_1]_B - k^B_{off,V164N1}[V_{164}N_1]_B \quad \text{(S.27)}$$
$$-k^B_{c,V164N1,R2}[V_{164}N_1]_B[R_2]_B + k^B_{off,V164N1R2}[R_2V_{164}N_1]_B$$

$$\frac{d[R_2V_{164}N_1]_B}{dt} = -k^B_{int,V164R2N1}[R_2V_{164}N_1]_B$$
$$+k^B_{c,V164R2,N1}[V_{164}R_2]_B[N_1]_B - k^B_{off,V164R2N1}[R_2V_{164}N_1]_B \quad \text{(S.28)}$$
$$+k^B_{c,V164N1,R2}[V_{164}N_1]_B[R_2]_B - k^B_{off,V164N1R2}[R_2V_{164}N_1]_B$$

$$\frac{d[V_{120}R_1]_B}{dt} = -k^B_{int,V120R1}[V_{120}R_1]_B$$
$$+k^B_{on,V120,R1}[V_{120}]_B[R_1]_B - k^B_{off,V120R1}[V_{120}R_1]_B \quad \text{(S.29)}$$
$$-k^B_{c,R1,N1}[V_{120}R_1]_B[N_1]_B + k^B_{dissoc,R1N1}[V_{120}R_1N_1]_B$$

$$\frac{d[V_{120}R_2]_B}{dt} = -k^B_{int,V120R2}[V_{120}R_2]_B + k^B_{on,V120,R2}[V_{120}]_B[R_2]_B - k^B_{off,V120R2}[V_{120}R_2]_B \tag{S.30}$$

$$\begin{aligned}\frac{d[R_1N_1]_B}{dt} = &-k^B_{int,R1N1}[R_1N_1]_B \\ &+k^B_{c,R1,N1}[R_1]_B[N_1]_B - k^B_{dissoc,R1N1}[R_1N_1]_B \\ &-k^B_{on,V120,R1}[V_{120}]_B[R_1N_1]_B + k^B_{off,V120R1}[V_{120}R_1N_1]_B\end{aligned} \tag{S.31}$$

$$\begin{aligned}\frac{d[V_{120}R_1N_1]_B}{dt} = &-k^B_{intV120R1N1}[V_{120}R_1N_1]_B \\ &+k^B_{c,V120R1,N1}[V_{120}R_1]_B[N_1]_B - k^B_{dissoc,V120N1}[V_{120}R_1N_1]_B \\ &+k^B_{on,V120R1N1}[V_{120}]_B[R_1N_1]_B - k^B_{off,V120R1N1}[V_{120}R_1N_1]_B\end{aligned} \tag{S.32}$$

$$\begin{aligned}\frac{d[N_1]_{N,myo}}{dt} = &s^{N,myo}_{N1} - k^{N,myo}_{\mathrm{int},N1}[N_1]_{N,myo} \\ &+k^{N,myo}_{on,V164,N1}[V_{164}]_N[N_1]_{N,myo} - k^{N,myo}_{off,V164N1}[V_{164}N_1]_{N,myo}\end{aligned} \tag{S.33}$$

$$\begin{aligned}\frac{d[V_{164}N_1]_{N.myo}}{dt} = &-k^{N,myo}_{int,V164N1}[V_{164}N_1]_{N,myo} \\ &+k^{N,myo}_{on,V164,N1}[V_{164}]_N[N_1]_{N,myo} - k^{N,myo}_{off,V164N1}[V_{164}N_1]_{N,myo}\end{aligned} \tag{S.34}$$

$$\begin{aligned}\frac{d[A]_B}{dt} = &q^B_A - c_A[A]_B - k^B_{on,V164,A}[V_{164}]_B[A]_B + k^B_{off,V164A}[V_{164}A]_B \\ &-k^B_{on,V120,A}[V_{120}]_B[A]_B + k^B_{off,V120A}[V_{120}A]_B \\ &+k^{BN}_{pA}\frac{S_{NB}}{U_p}[A]_B - \left(\frac{k_L + k^{NB}_{pA}S_{NB}}{U_B}\right)\frac{[A]_N}{K_{AV,N}}\end{aligned} \tag{S.35}$$

$$\begin{aligned}\frac{d[V_{164}A]_B}{dt} = &-c_{V164A}[V_{164}A]_B + k^B_{on,V164,A}[V_{164}]_B[A]_B - k^B_{off,V164A}[V_{164}A]_B \\ &-k^{BN}_{pA}\frac{S_{NB}}{U_p}[V_{164}A]_B + \left(\frac{k_L + k^{NB}_{pA}S_{NB}}{U_B}\right)\frac{[V_{164}A]_N}{K_{AV,N}}\end{aligned} \tag{S.36}$$

$$\begin{aligned}\frac{d[V_{120}A]_B}{dt} = &-c_{V120A}[V_{120}A]_B + k^B_{on,V120,A}[V_{120}]_B[A]_B - k^B_{off,V120A}[V_{120}A]_B \\ &-k^{BN}_{pA}\frac{S_{NB}}{U_p}[V_{120}A]_B + \left(\frac{k_L + k^{NB}_{pA}S_{NB}}{U_B}\right)\frac{[V_{120}A]_N}{K_{AV,N}}\end{aligned} \tag{S.37}$$

$$\frac{d[A]_N}{dt} = -k^N_{on,V164,A}[V_{164}]_N[A]_N + k^N_{off,V164A}[V_{164}A]_N$$
$$-k^N_{on,V120,A}[V_{120}]_N[A]_N + k^N_{off,V120A}[V_{120}A]_N \tag{S.38}$$
$$+k^{BN}_{pA}\frac{S_{NB}}{U_N}\frac{U_B}{U_P}[A]_B - \left(\frac{k_L + k^{NB}_{pA}S_{NB}}{U_N}\right)\frac{[A]_N}{K_{AV,N}}$$

$$\frac{d[V_{164}A]_N}{dt} = k^N_{on,V164,A}[V_{164}]_N[A]_N - k^N_{off,V164A}[V_{164}A]_N$$
$$+k^{BN}_{pA}\frac{S_{NB}}{U_N}\frac{U_B}{U_p}[V_{164}A]_B - \left(\frac{k_L + k^{NB}_{pA}S_{NB}}{U_N}\right)\frac{[V_{164}A]_N}{K_{AV,N}} \tag{S.39}$$

$$\frac{d[V_{120}A]_N}{dt} = k^N_{on,V120,A}[V_{120}]_N[A]_N - k^N_{off,V120A}[V_{120}A]_N$$
$$+k^{BN}_{pA}\frac{S_{NB}}{U_N}\frac{U_B}{U_p}[V_{120}A]_B - \left(\frac{k_L + k^{NB}_{pA}S_{NB}}{U_N}\right)\frac{[V_{120}A]_N}{K_{AV,N}} \tag{S.40}$$

Glossary

Concentrations

$[V_{120}]$, $[V_{164}]$	Concentration of unbound $VEGF_{120}$ and $VEGF_{164}$
$[M_{ECM}]$, $[M_{EBM}]$, $[M_{PBM}]$	Concentration of VEGF binding sites in the ECM, EBM, and PBM
$[V_{164}M_{ECM}]$, $[V_{164}M_{EBM}]$, $[V_{164}M_{PBM}]$	Concentration of $VEGF_{164}$ bound to the ECM, EBM, and PBM
$[R_1]$, $[R_2]$	Concentration of un-occupiedVEGFR-1 and VEGFR-2 receptor tyrosine kinases
$[N_1]$	Concentration of un-occupied NRP-1 co-receptor (on endothelial cells)
$[N_1]_{myo}$	Concentration of un-occupied NRP-1 co-receptor (on myocytes)
$[R_1N_1]$	Concentration of the VEGFR-1-NRP-1 complex
$[V_iR_j]$	Concentration of VEGF isoform *i* bound to VEGFR *j*
$[V_iN_1]$	Concentration of VEGF isoform *i* bound to NRP-1
$[R_2V_{164}N_1]$	Concentration of the VEGFR-2-$VEGF_{164}$-NRP-1 ternary complex
$[V_{120}R_1N_1]$	Concentration of the $VEGF_{120}$-VEGFR-1-NRP-1 ternary complex
$[V_{164}N_1]_{myo}$	Concentration of $VEGF_{164}$ bound to NRP-1 (on myocytes)
$[A]$	Concentration of anti-VEGF agent

$[V_iA]$	Concentration of VEGF isoform *i* bound to anti-VEGF agent

Geometric parameters

U_i	Volume of compartment *i* (N=tissue, B=blood, P=plasma)
S_{NB}	Total surface area of endothelial cells at the interface of tissue (N) and blood (B)
$K_{AV,i}$	Available volume fraction in the tissue, i.e., ratio of available fluid volume to total tissue volume U_i

Kinetic parameters

q_{V120}, q_{V164}	Secretion rate of $VEGF_{120}$ and $VEGF_{164}$
q_A	Injection rate of anti-VEGF agent
s_R	Insertion rate of receptors into the cell membrane of endothelial cells or myocytes
k_{on}	Kinetic binding rate
k_{off}	Kinetic unbinding rate
k_c	Kinetic coupling rate for receptors
k_{int}	Internalization rate of receptors
k_{pV}^{ij}	Microvascular permeability of VEGF from compartment *i* to compartment *j* (N=tissue, B=blood)
k_{pA}^{ij}	Microvascular permeability of anti-VEGF agent and

	VEGF/anti-VEGF complex from compartment i to compartment j (N=tissue, B=blood)
k_L	Lymphatic drainage rate
c_{V120}, c_{V164}	Rate of plasma clearance of $VEGF_{120}$ and $VEGF_{164}$
c_A, c_{V120A}, c_{V164A}	Rate of plasma clearance of anti-VEGF and VEGF/anti-VEGF complex
