# Supplementary material for: A Two-Compartment Model of VEGF Distribution in the Mouse
Source: PLoS One. 2011 Nov 8;6(11):e27514. doi: 10.1371/journal.pone.0027514 (PMC3210788; doi:10.1371/journal.pone.0027514)
Supplement: Table S1 — Optimized parameter values from twenty optimization trials. (DOC) [file pone.0027514.s002.doc]

***Table S1: Parameter optimization results***

| Trial | WSSR | *q_V164_* (molecules/cell/s) | *k_L_* (cm^3^/s) | *c_A_* (x 10^-4^ min^-1^) | *c_VA_* (x 10^-4^ min^-1^) | *K_d_* (pM) |
| --- | --- | --- | --- | --- | --- | --- |
| 1 | 8.137 | 0.0626 | 7.00 x 10^-6^ | 8.86 | 2.79 | 0.37 |
| 2 | 8.138 | 0.0627 | 7.04 x 10^-6^ | 8.85 | 2.80 | 0.37 |
| 3 | 8.139 | 0.0627 | 7.00 x 10^-6^ | 8.83 | 2.81 | 0.37 |
| 4 | 8.139 | 0.0629 | 7.07 x 10^-6^ | 8.83 | 2.84 | 0.37 |
| 5 | 8.140 | 0.0625 | 7.17 x 10^-6^ | 8.85 | 2.79 | 0.37 |
| 6 | 8.141 | 0.0624 | 7.14 x 10^-6^ | 8.90 | 2.74 | 0.38 |
| 7 | 8.141 | 0.0620 | 7.00 x 10^-6^ | 8.92 | 2.72 | 0.38 |
| 8 | 8.142 | 0.0628 | 7.25 x 10^-6^ | 8.82 | 2.82 | 0.37 |
| 9 | 8.148 | 0.0619 | 7.58 x 10^-6^ | 8.88 | 2.73 | 0.38 |
| 10 | 8.156 | 0.0629 | 8.23 x 10^-6^ | 8.85 | 2.74 | 0.38 |
| 11 | 8.162 | 0.0643 | 7.41 x 10^-6^ | 8.67 | 2.98 | 0.36 |
| 12 | 8.174 | 0.0613 | 1.01 x 10^-5^ | 8.80 | 2.69 | 0.37 |
| 13 | 8.176 | 0.0609 | 8.14 x 10^-6^ | 9.06 | 2.51 | 0.41 |
| 14 | 8.176 | 0.0609 | 8.14 x 10^-6^ | 9.06 | 2.51 | 0.41 |
| 15 | 8.182 | 0.0638 | 7.56 x 10^-6^ | 8.60 | 3.07 | 0.34 |
| 16 | 8.201 | 0.0595 | 1.23 x 10^-5^ | 8.83 | 2.54 | 0.37 |
| 17 | 8.244 | 0.0647 | 8.85 x 10^-6^ | 8.45 | 3.20 | 0.32 |
| 18 | 8.258 | 0.0618 | 1.48 x 10^-5^ | 8.45 | 2.82 | 0.35 |
| 19 | 8.288 | 0.0628 | 7.06 x 10^-6^ | 9.38 | 2.27 | 0.48 |
| 20 | 8.382 | 0.0544 | 2.73 x 10^-5^ | 8.53 | 2.16 | 0.35 |
| min | 8.137 | 0.0544 | 7.00 x 10^-6^ | 8.45 | 2.16 | 0.32 |
| max | 8.382 | 0.0647 | 2.73 x 10^-5^ | 9.38 | 3.20 | 0.48 |

WSSR: weighted sum of squared residuals
