# Supplementary material for: A Two-Compartment Model of VEGF Distribution in the Mouse
Source: PLoS One. 2011 Nov 8;6(11):e27514. doi: 10.1371/journal.pone.0027514 (PMC3210788; doi:10.1371/journal.pone.0027514)
Supplement: Table S2 — Optimized parameter values from twenty optimization trials when VEGF degradation is included. (DOC) [file pone.0027514.s003.doc]

***Table S2: Parameter optimization results when VEGF degradation is included***

| Trial | WSSR | *q_V164_* (molecules/cell/s) | *k_L_* (cm^3^/s) | *c_A_* (x 10^-4^ min^-1^) | *c_VA_* (x 10^-4^ min^-1^) | *K_d_* (pM) |
| --- | --- | --- | --- | --- | --- | --- |
| 1 | 8.166 | 0.0631 | 7.01 x 10^-6^ | 8.79 | 2.85 | 0.36 |
| 2 | 8.183 | 0.0622 | 8.53 x 10^-6^ | 8.83 | 2.74 | 0.37 |
| 3 | 8.163 | 0.0626 | 7.00 x 10^-6^ | 8.85 | 2.79 | 0.37 |
| 4 | 8.186 | 0.0604 | 7.00 x 10^-6^ | 9.09 | 2.54 | 0.39 |
| 5 | 8.221 | 0.0644 | 8.87 x 10^-6^ | 8.55 | 3.02 | 0.35 |
| 6 | 8.162 | 0.0622 | 7.00 x 10^-6^ | 8.91 | 2.73 | 0.38 |
| 7 | 8.195 | 0.0627 | 9.18 x 10^-6^ | 8.70 | 2.85 | 0.36 |
| 8 | 9.744 | 0.0312 | 1.60 x 10^-4^ | 7.67 | 0.39 | 0.29 |
| 9 | 8.161 | 0.0627 | 7.00 x 10^-6^ | 8.85 | 2.79 | 0.37 |
| 10 | 8.164 | 0.0632 | 7.02 x 10^-6^ | 8.82 | 2.83 | 0.37 |
| 11 | 8.167 | 0.0617 | 7.24 x 10^-6^ | 8.90 | 2.72 | 0.37 |
| 12 | 8.162 | 0.0621 | 7.00 x 10^-6^ | 8.85 | 2.78 | 0.37 |
| 13 | 8.637 | 0.0482 | 4.59 x 10^-5^ | 8.24 | 1.79 | 0.31 |
| 14 | 8.165 | 0.0633 | 7.00 x 10^-6^ | 8.78 | 2.86 | 0.37 |
| 15 | 8.164 | 0.0623 | 7.12 x 10^-6^ | 8.87 | 2.78 | 0.37 |
| 16 | 8.164 | 0.0624 | 7.00 x 10^-6^ | 8.91 | 2.74 | 0.38 |
| 17 | 8.165 | 0.0628 | 7.06 x 10^-6^ | 8.85 | 2.80 | 0.37 |
| 18 | 8.166 | 0.0617 | 7.04 x 10^-6^ | 8.88 | 2.73 | 0.37 |
| 19 | 8.168 | 0.0627 | 7.06 x 10^-6^ | 8.87 | 2.77 | 0.37 |
| 20 | 8.412 | 0.0563 | 2.71 x 10^-5^ | 8.34 | 2.38 | 0.33 |
| min | 8.161 | 0.0312 | 7.00 x 10^-6^ | 7.67 | 0.39 | 0.29 |
| max | 9.744 | 0.0644 | 1.60 x 10^-4^ | 9.09 | 3.02 | 0.39 |

WSSR: weighted sum of squared residuals
